# Mosquito species identification using convolutional neural networks with a multitiered ensemble model for novel species detection

**DOI:** 10.1038/s41598-021-92891-9

**Published:** 2021-07-01

**Authors:** Autumn Goodwin, Sanket Padmanabhan, Sanchit Hira, Margaret Glancey, Monet Slinowsky, Rakhil Immidisetti, Laura Scavo, Jewell Brey, Bala Murali Manoghar Sai Sudhakar, Tristan Ford, Collyn Heier, Yvonne-Marie Linton, David B. Pecor, Laura Caicedo-Quiroga, Soumyadipta Acharya

**Affiliations:** 1 Vectech, Baltimore, MD 21211 USA; 2 Center for Bioengineering Innovation and Design, Biomedical Engineering Department, Whiting School of Engineering, The Johns Hopkins University, Baltimore, MD 21218 USA; 3 The Laboratory for Computational Sensing and Robotics, Whiting School of Engineering, The Johns Hopkins University, Baltimore, MD 21218 USA; 4 Walter Reed Biosystematics Unit (WRBU), Smithsonian Institution Museum Support Center, Suitland, MD 20746 USA; 5 Walter Reed Army Institute of Research, Silver Spring, MD 20910 USA; 6 Department of Entomology, Smithsonian Institution-National Museum of Natural History, Washington, DC 20560 USA

**Keywords:** Biodiversity, Ecological modelling, Population screening, Computer science

## Abstract

With over 3500 mosquito species described, accurate species identification of the few implicated in disease transmission is critical to mosquito borne disease mitigation. Yet this task is hindered by limited global taxonomic expertise and specimen damage consistent across common capture methods. Convolutional neural networks (CNNs) are promising with limited sets of species, but image database requirements restrict practical implementation. Using an image database of 2696 specimens from 67 mosquito species, we address the practical open-set problem with a detection algorithm for novel species. Closed-set classification of 16 known species achieved 97.04 ± 0.87% accuracy independently, and 89.07 ± 5.58% when cascaded with novelty detection. Closed-set classification of 39 species produces a macro F1-score of 86.07 ± 1.81%. This demonstrates an accurate, scalable, and practical computer vision solution to identify wild-caught mosquitoes for implementation in biosurveillance and targeted vector control programs, without the need for extensive image database development for each new target region.

## Introduction

Mosquitoes are one of the deadliest animals in the world, infecting between 250–500 million people every year with a wide range of fatal or debilitating diseases, including malaria, dengue, chikungunya, Zika and West Nile Virus^1^. Mosquito control programs, currently the most effective method to prevent mosquito borne disease, rely on accurate identification of disease vectors to deploy targeted interventions, which reduce populations and inhibit biting, lowering transmission^2^. Vector surveillance—assessing mosquito abundance, distribution, and species composition data—is critical to both optimizing intervention strategies and evaluating their efficacy^2,3^. Despite advances in vector control, mosquito borne disease continues to cause death and debilitation, with climate change^4^, insecticide resistance^5^, and drug resistant pathogens^6,7^ cited as exacerbating factors.

Accurate mosquito identification is essential, as disparate taxa exhibit distinct biologies (e.g. host preference, biting patterns), and innate immunities, which influence vector competencies and inform effective intervention strategies^3^. Typically, mosquito surveillance requires the microscopic screening of thousands of mosquitoes per week and identification to species using dichotomous keys^8–10^—a technical, time-intensive activity that is difficult to scale. One of the most substantial barriers to effective biosurveillance activities is the delayed data-to-decision pathway, caused by the identification bottlenecks centered on severely limited global expertise in insect identification^11,12^. In order to address this global taxonomic impediment^13^ and to increase the pace of reliable mosquito species identification, several automated methods have been explored, including analysis of wingbeat frequencies^14,15^, traditional computer vision approaches^16^, and most notably using deep Convolutional Neural Networks (CNNs) to recognize mosquito images^17–20^.

Computer vision holds promise as a scalable method for reliable identification of mosquitoes, given that—like mosquito taxonomists—it relies on visual (morphological) characters to assign a definitive taxon name. Using CNNs, Park et al*.* showed an impressive 97% accuracy over eight species^19^, two of which were sourced from lab-reared colonies. However, morphologically similar species were grouped together, with all species from the genus *Anopheles* considered as one class^19^ [herein, a class is described as a distinct output category of a machine learning model for a classification task]. Couret et al*.* achieved 96.96% accuracy with CNNs amongst 17 classes^20^, including differentiation of closely related cryptic species in the *Anopheles gambiae* species complex as independent classes. For ease of gathering mosquito specimens for acquiring image data, both studies relied heavily on available colony strains. This invokes criticism of the field utility of this approach by mosquito workers as (1) species comparisons used do not reflect naturally occurring biodiversity in a given location, (2) inbred colonies can quickly lose or fix unique phenotypes that are not maintained in field caught specimens, and (3) the colony material is pristine compared to the battered samples typically collected using suction traps in the field, raising doubts as to whether the technology is field transferrable. Nonetheless these initial studies have shown the potential for CNNs to automate species identification, which, if expanded and based on expertly identified material, would facilitate near real-time vector control and critical biosurveillance activities across the globe.

While previous works^17–20^ have proven the utility of deep learning for recognizing mosquito species, they have not addressed the substantial impediments caused by field-collected specimens. In real-world scenarios, identification is a noisy problem amplified by high inter- and intra-specific diversity, and by the recovery of aged, often damaged specimens missing key identifying features (scales, setae) or body parts during trapping, storage (dry/frozen) and transportation. Addressing the increased complexity of identifying wild-caught mosquito specimens is essential for developing a practical computer vision solution usable by mosquito control programs. Mosquito identification can be challenging, with many species of similar morphology. While some previous work has gone beyond genus-level classification to identify species, most have tested morphologically dissimilar species^19^, or pristine specimens readily available from laboratory colonies^20^, rather than environmental specimens.

Reliance on laboratory reared specimens aside, all computer vision methods for mosquito classification to date have been limited to closed-set classification of no more than 17 species. There are currently 3570 formally described species and 130 subspecies in the 41 genera that comprise the family Culicidae^21^, and many new species detected genetically that await formal descriptions. Given this, a close-to-complete dataset of all mosquitoes is unlikely and any computer vision solution must be able to recognize when it encounters a species not represented in the closed-set of species as previously unencountered, rather than forcing incorrect species assignment: an open set recognition, or novelty detection, problem. The novelty detection and rejection problem has been addressed in previous computer vision work, though most successful methods are applied to non-fine-grained problems represented by ImageNet^22^, CIFAR^23^, and MNIST^24^ datasets^25^, such as the generalized out-of-distribution image detection (ODIN) of Hsu et al.^26^ and multi-task learning for open set recognition of Oza et al.^27^.

**Figure 1:**
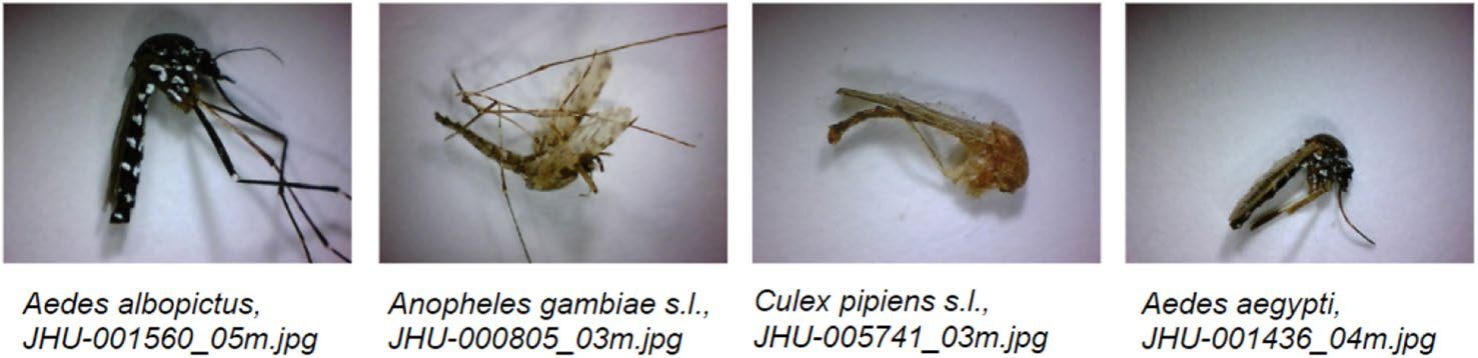
Example image database samples of wild-caught mosquitoes. Field specimens were often considerably damaged, missing appendages, scales, and hairs, like specimens captured during standard mosquito surveillance practice.

Given the fine-grained morphological diversity between closely related mosquito species and the intra-specific variability (of both form and acquired damage) between wild-caught specimens, this problem is succinctly defined as a noisy, fine-grained, open set recognition object classification problem. Defining the problem as such is necessary for practical implementation, and has not yet been addressed, nor has this type of problem been sufficiently solved in the wider computer vision literature. Herein, we present a convolutional neural network-based approach to open set recognition, designed for identifying wild caught mosquito specimens. When presented with an image, our algorithm first determines whether its species is known to the system, or is novel. If known, it classifies the specimen as one of the known species. The novelty detection algorithm was evaluated using 25-fold cross validation over 67 species, and the final classification CNN using fivefold cross-validation over 20 potentially known species, using 16 of these species as known for each fold, such that each known species is considered unknown for one of the five folds of the CNNs. The data used for evaluation included species which can be devised into three distinct groups: (1) known species, which the closed-set CNN is trained on for species classification; (2) unknown species used in training, which are used to train other components of the novelty detection algorithm to distinguish unknown species from known species; and (3) novel unknown species, which are not used in training and are completely novel to the CNN. Thus we test a representation of the real-world practical use of such an algorithm. All specimens used in the study were expertly identified (by morphology or by DNA barcoding), thus reference to known or unknown species in this context is indicative of whether the CNN has prior knowledge of the species presented. We also provide an evaluation of 39 species closed-set classification and a comparison of our methods to some common alternatives. The image database developed for training and testing this system consists of primarily wild caught specimens bearing substantial physical damage, obfuscating some diagnostic characters (see Fig. 1).

## Results

### System overview

To address the open-set novel species detection problem, our system leverages a two-step image recognition process. Given an image of a mosquito specimen, the first step uses CNNs trained for species classification to extract relevant features from the image. The second step is a novelty detection algorithm, which evaluates the features extracted by the CNNs in order to detect whether the mosquito is a member of one of the sixteen species known to the CNNs of the system. The second step consists of two stages of machine learning algorithms (tier II and tier III) that evaluate the features generated in step one to separate known species from unknown species. Tier II components evaluate the features directly and are trained using known and unknown species. Tier III evaluates the answers provided by the tier II components to determine the final answer, and is trained using known species, unknown species used for training tier II components, and still more unknown species not seen by previous components. If the mosquito is determined by tier III not to be a member of one of the known species, it is classified as an unknown species, novel to the CNNs. This detection algorithm is tested on truly novel mosquito species, never seen by the system in training, as well as the species used in training. If a mosquito is recognized by the system as belonging to one of the sixteen known species (i.e. not novel), the image proceeds to species classification with one of the CNNs used to extract features.

### Unknown detection accuracy

In distinguishing between unknown species and known species, the algorithm achieved an average accuracy of 89.50 ± 5.63% and 87.71 ± 2.57%, average sensitivity of 92.18 ± 6.34% and 94.09 ± 2.52%, and specificity of 80.79 ± 7.32% and 75.82 ± 4.65%, micro-averaged and macro-averaged respectively, evaluated over twenty-five-fold validation (Table 1). Here, micro-average refers to the metric calculated without regard to species, such that each image sample has an equal weight, considered an image sample level metric. Macro-average refers to the metric first calculated within a species, then averaged between all species within the relevant class (known or unknown). Macro-average can be considered a species level metric, or a species normalized metric. Macro-averages tend to be lower than the micro-averages when species with higher sample sizes have the highest metrics, whereas micro-averages are lower when species with lower sample sizes have the highest metrics. Cross validation by mixing up which species were known and unknown produced variable sample sizes in each iteration, because each species had a different number of samples in the generated image dataset. Further sample size variation occurred as a result of addressing class imbalance in the training set. The mean number of samples varied for each of the 25 iterations because of the mix-up in data partitioning for cross-validation (see Table 1 for generalized metrics; see Supplementary Table 1, Datafolds for detailed sampling data).

**Table 1:** Micro- and macro-averaged metrics of the novelty detection algorithm on the test set using 50-fold validation.

	Known samples (*avg* (*min–max*))	Unknown samples (*avg* (*min–max*))	Average accuracy	Unknown sensitivity/recall	Unknown specificity	Unknown precision
Micro-average ^a^	519.44 (409–694)	2216.12 (1043–3557)	89.50 ± 5.63%	92.18 ± 6.34%	80.79 ± 7.32%	94.21 ± 3.76%
Macro-average ^b^	15.8 (15–16)^c^	29.84 (25–34)^c^	87.71 ± 2.57%	94.09 ± 2.52%	75.82 ± 4.65%	79.66 ± 3.24%

^a^Micro-average refers to the metric calculated without regard to species as an independent class: each sample has an equal weight.

^b^Macro-average refers to the metric calculated first within each species as an independent class, then averaged between species for the known or unknown classes, in order to address imbalance of samples between species.

^c^This indicated the number of species in the known or unknown classes for each macro-average.

### Differences within the unknown species dictated by algorithm structure

The fundamental aim of novelty detection is to determine if the CNN in question is familiar with the species, or class, shown in the image. CNNs are designed to identify visually distinguishable classes, or categories. In our open-set problem, the distinction between known and unknown species is arbitrary from a visual perspective; it is only a product of the available data. However, the known or unknown status of a specimen is a determinable product of the feature layer outputs, or features, produced by the CNN’s visual processing of the image. Thus, we take a tiered approach, where CNNs trained on a specific set of species extract a specimen’s features, and independent classifiers trained on a wider set of species analyze the features produced by the CNNs to assess whether the CNNs are familiar with the species in question. The novelty detection algorithm consists of three tiers, hereafter referred to as Tier I, II, and III, intended to determine if the specimen being analyzed is from a closed set of species known to the CNN:

Tier I: two CNNs used to extract features from the images.

Tier II: a set of classifiers, such as SVMs, random forests, and neural networks, which independently process the features from Tier I CNNs to distinguish a specimen as either known or unknown species.

Tier III: soft voting of the Tier II classifications, with a clustering algorithm, in this case a Gaussian Mixture Model (GMM), which is used to make determinations in the case of unconfident predictions.

**Figure 2:**
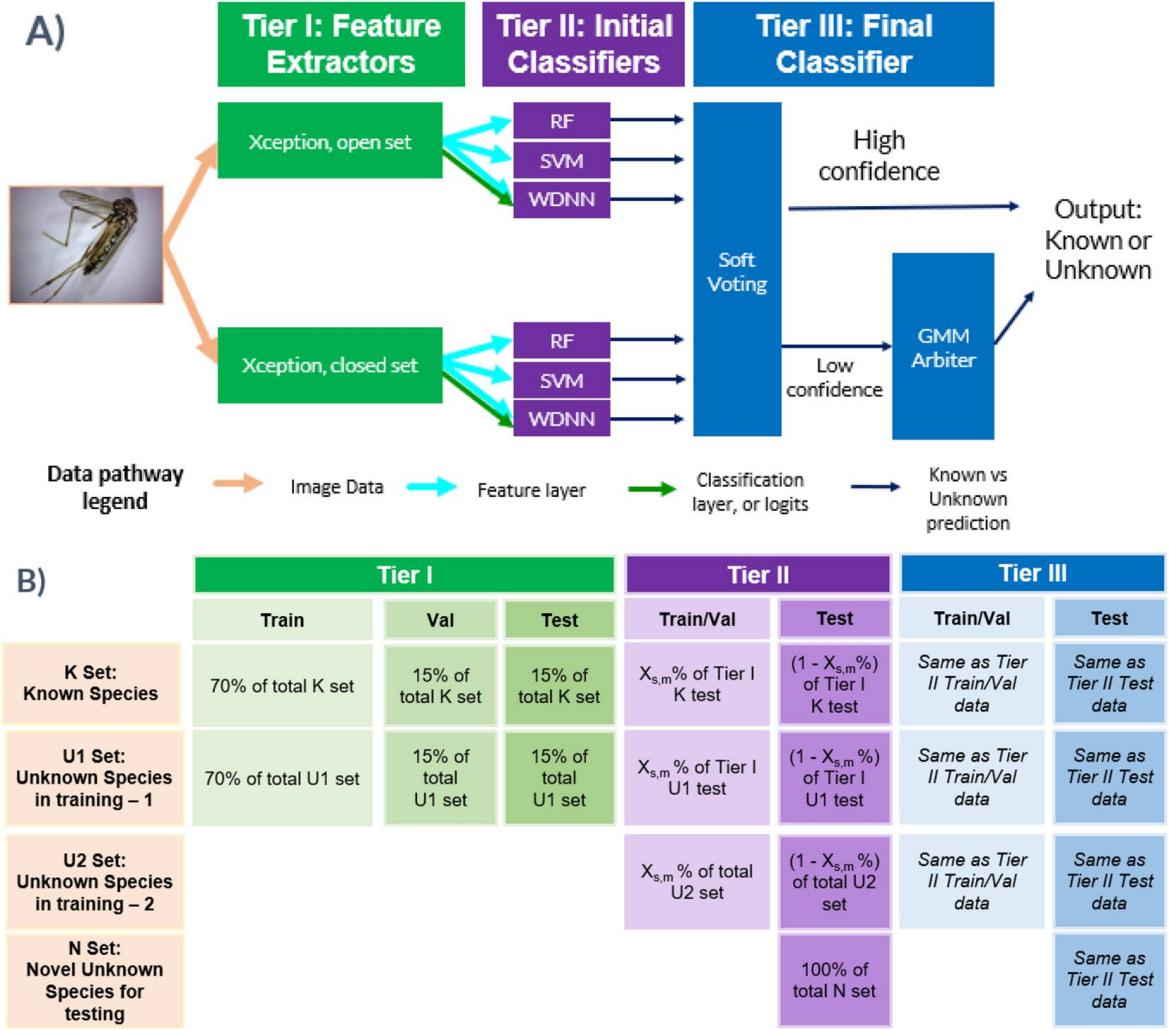
The novelty detection architecture was designed with three tiers to assess whether the CNNs were familiar with the species shown in each image. (**A**) Tier I consisted of two CNNs used as feature extractors. Tier II consisted of initial classifiers making an initial determination about whether the specimen is known or unknown by analyzing the features of one of the Tier I CNNs, and the logits in the case of the wide and deep neural network (WDNN). In this figure, SVM refers to a support vector machine, and RF refers to a random forest. Tier III makes the final classification, first with soft voting of the Tier II outputs, then sending high confidence predictions as the final output and low confidence predictions to a Gaussian Mixture Model (GMM) to serve as the arbiter for low confidence predictions. (**B**) Data partitioning for training each component of the architecture is summarized: Tier I is trained on the K set of species, known to the algorithm; Tier I open-set CNN is also trained on the U1 set of species, the first set of unknown species used in training; Tier II is trained on K set, U1 set, and the U2 set of species, the second set of unknown species used in training; Tier III is trained on the same species and data-split as Tier II. Data-split ratios were variable for each species over each iteration (X_s,m_ where *s* represents a species, *m* represents a fold, and X is a percentage of the data devoted to training) for Tiers II and III; X_s,m_ was adjusted to manage class imbalance within genus across known and unknown classes. Testing was performed on each of the K, U1, and U2 sets, as well as the N set, the final set of unknown species reserved for testing the algorithm, such that it is tested on previously unseen taxa, replicating the plausible scenario to be encountered in deployment of CNNs for species classification. Over the twenty-five folds, each known species was considered unknown for at least five folds and included as novel for at least one-fold.

The tiered architecture necessitated partitioning of groups of species between the tiers, and an overview of the structure is summarized in Fig. 2A. The training schema resulted in three populations of unknown species: set U1, consisting of species used to train Tier I, also made available for training subsequent Tiers II and III; set U2, consisting of additional species unknown to the CNNs used to train Tiers II and III; and set N, consisting of species used only for testing (see Fig. 2B). Species known to the CNNs are referred to as set K. It is critical to measure the difference between these species sets, as any of the species may be encountered in the wild. U1 achieved 97.85 ± 2.81% micro-averaged accuracy and 97.34 ± 3.52% macro-averaged accuracy; U2 achieved 97.05 ± 1.94% micro-averaged accuracy and 97.30 ± 1.41% macro-averaged accuracy; N achieved 80.83 ± 19.91% micro-averaged accuracy and 88.72 ± 5.42% macro-averaged accuracy. The K set achieved 80.79 ± 7.32% micro-averaged accuracy and 75.83 ± 5.42% macro-averaged accuracy (see Table 2). The test set sample sizes for each of the twenty five folds are as follows, (formatted [K-taxa,K-samples;U1-taxa,U1-samples;U2-taxa,U2-samples;N-taxa,N-samples]): [16,683;8,51;10,536;13,456], [16,673;8,51;9,537;13,485], [16,673;8,51;8,523;13,508], [16,673;8,46;6,159;11,869], [16,694;8,51;7,483;10,548], [15,409;9,62;11,2906;8,546], [15,456;9,62;9,2458;12,1024], [15,456;10,67;13,2359;9,1115], [15,456;9,62;8,3189;12,306], [15,456;10,67;10,2874;10,601], [16,543;10,56;12,1450;10,1052], [16,484;9,52;11,2141;10,312], [16,492;10,54;11,2185;12,263], [16,512;8,45;15,2292;10,189], [16,480;9,49;9,1652;13,790], [16,442;9,44;11,1253;11,665], [16,494;10,54;14,1727;10,228], [16,442;9,55;13,1803;10,96], [16,538;10,60;8,1509;9,502], [16,489;10,60;13,1764;9,184], [16,462;8,47;13,1415;11,452], [16,437;8,54;9,1548;11,320], [16,447;8,55;11,654;10,1193], [16,547;8,44;9,1437;11,531], [16,548;7,52;7,1464;11,499]. See Supplementary Table 1, Datafolds for more detailed sample information.

**Table 2:** Accuracy metrics for the known, unknown, and novel unknown species sets over twenty-five-fold validation.

	Accuracy, macro-average	Accuracy, micro-average	Number of species tested within set (*avg (min–max)*)^b^	Number of samples within set (*avg (min–max)*)^b^
Set K^a^	75.83 ± 4.65%	80.79 ± 7.32%	15.8 (15–16)	519.44 (409–694)
Set U1^a^	97.34 ± 3.52%	97.85 ± 2.81%	8.8 (7–10)	54.04 (44–67)
Set U2^a^	97.30 ± 1.41%	97.05 ± 1.94%	10.28 (6–15)	1612.72 (159–3189)
Set N^a^	88.72 ± 5.42%	80.83 ± 19.91%	10.76 (8–13)	549.36 (96–1193)

^a^Set K species are known to the closed-set CNNs. Set U1 unknown species are used in training for Tiers I, II & III. Set U2 unknown species are used to train Tier II&III. Set N unknown species are novel to all tiers and reserved for testing.

^b^The extreme variation in the test set was due to class balancing the Tier II&III training data, such that whenever a genus was represented in the K set a similar number of specimens from that genus were available for the unknown training set, whenever possible. Some of the U2 species were only used during training due to limited numbers of specimens in particular species.

### Subsequent species classification

**Figure 3:**
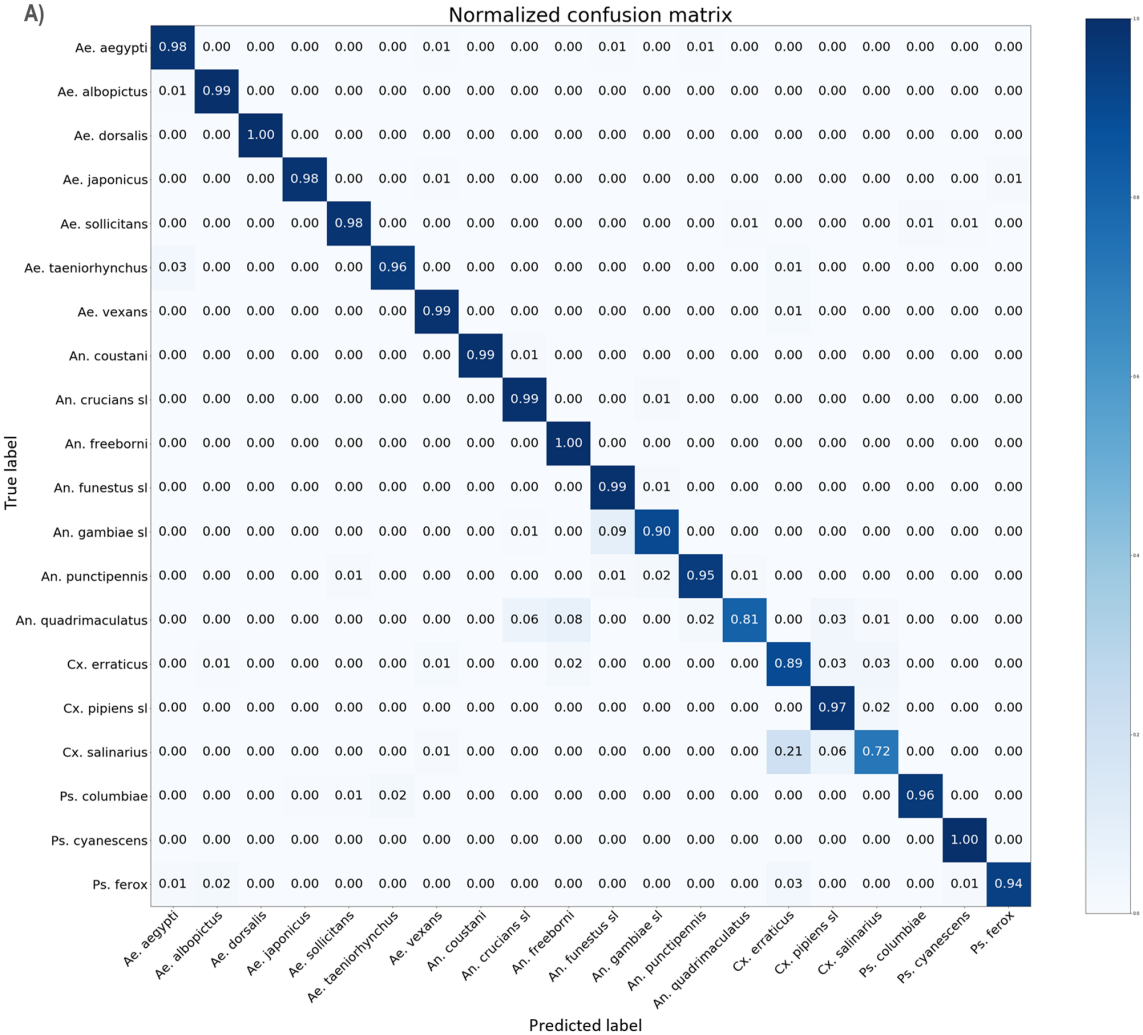

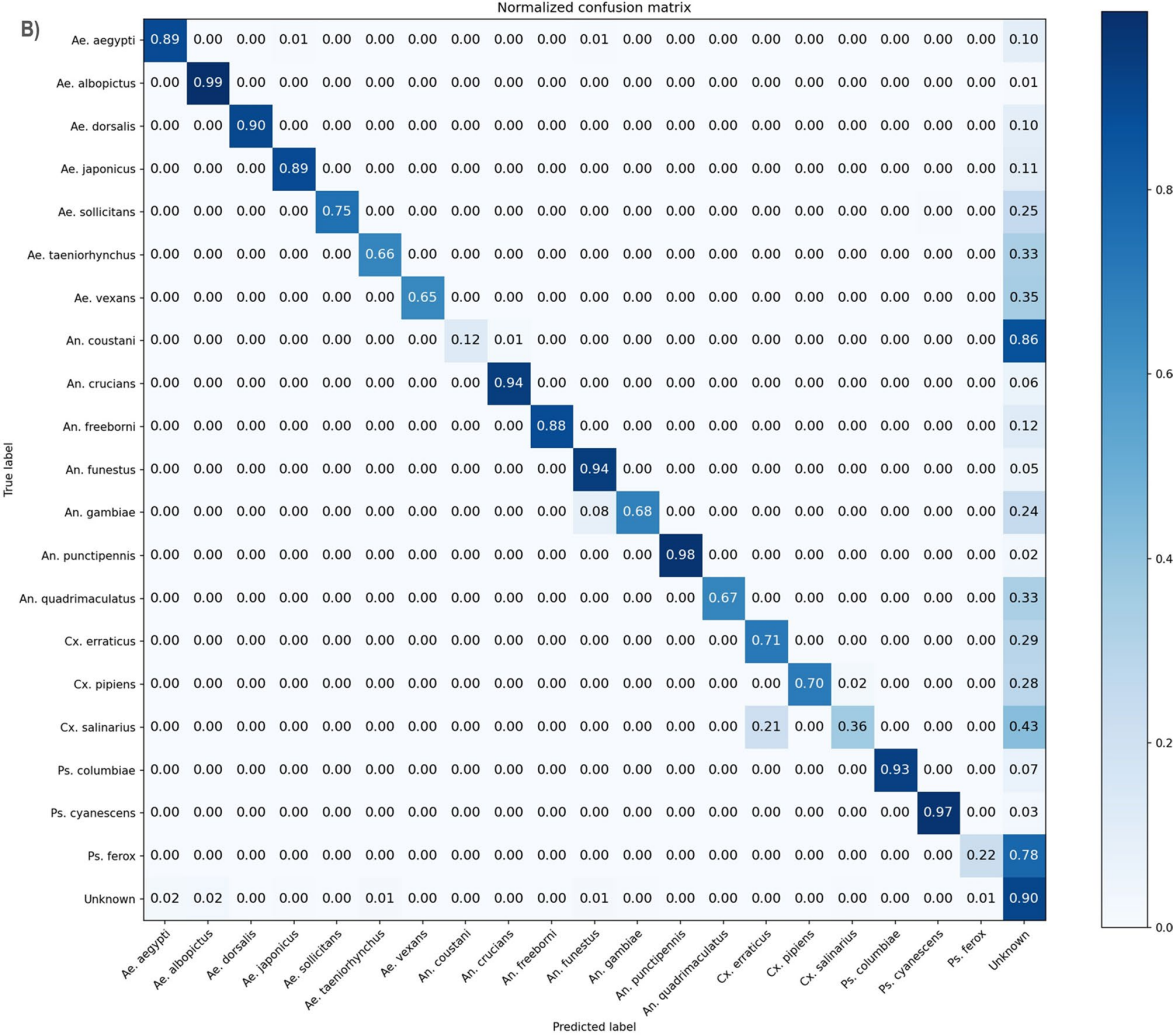
Mean normalized confusion matrices for species classification shows the distribution of error within species. The species classification in these confusion matrices was performed by the Tier I CNN, the closed-set Xception model. The confusion matrix conveys the ground truth of the sample horizontally, labels on the left, and the prediction of the full methods vertically, labels on the bottom. Accurate classification is across the diagonal, where ground truth and prediction match, and all other cells on the matrix describe the error. Sixteen species were known for a given fold, and 51 species were considered unknown for a given fold, with each of the twenty known species considered unknown for one fold. (**A**) The species classification independent of novelty detection shows an average accuracy of 97.04 ± 0.87% and a macro F1-score of 96.64 ± 0.96%, calculated over the five folds of Tier I classifiers, trained and tested over an average of 7174.8 and 1544.6 samples. Of the error, 73.5% occurred with species of the same genus as the true species. (**B**) The species classification as a subsequent step after novelty detection yielded 89.07 ± 5.58% average accuracy, and a macro F1-score of 79.74 ± 3.65% trained and tested on an average of 7174.8 and 519.44 samples, evaluated over the twenty-five folds of the novelty detection methods. First, a sample was sent to the novelty detection algorithm. If the sample was predicted to be known to the species classifier, which was the closed-set Xception algorithm used in Tier I, then the sample was sent to the algorithm for classification.

Following the novelty detection algorithm, species identified as known are sent for species classification to the closed-set Xception model used in Tier I of the novelty detection algorithm. Figure 3A shows the species classification results independently over the five folds of Tier I, which achieved a micro-averaged accuracy 97.04 ± 0.87% and a macro F1-score of 96.64 ± 0.96%. Figure 3B shows the species classification cascaded with the novelty detection methods where all unknown species are grouped into a single unknown class alongside the known classes in an aggregated mean confusion matrix over the twenty-five folds of the full methods, yielding a micro-averaged accuracy of 89.07 ± 5.58%, and a macro F1-score of 79.74 ± 3.65%. The confusion matrix is normalized by species and shows the average classification accuracy and error distribution. The independent accuracy for classifying a single species ranged from 72.44 ± 13.83% (*Culex salinarius*) to 100 ± 0% (*Aedes dorsalis, Psorophora cyanescens*), and 15 of the 20 species maintained an average sensitivity above 95%. Test set sample size for each species were as follows (formatted as species, [fold1,fold2,fold3,fold4,fold5]): *Ae. aegypti:* [127,0,133,132,126]; *Ae. albopictus:* [103,90,0,99,102]; *Ae. dorsalis:* [43,41,42,0,41]; *Ae. japonicus*: [162,159,154,156,0]; *Ae. sollicitans:* [57,0,60,58,60]; *Ae. taeniorhynchus:* [0,25,27,25,24]; *Ae. vexans:* [50,48,0,46,49]; *An. coustani:* [29,21,18,0,22]; *An. crucians* s.l.*: [*56,58,61,61,0]*; An. freeborni:* [87,0,77,79,80]; *An. funestus* s.l.*:* [158, 174,0,173,175]; *An. gambiae* s.l.*:* [182,178,178,0,166]; *An. punctipennis:* [0,36,31,34,33]; *An. quadrimaculatus:* [0,28,28,28,30]; *Cx. erraticus:* [47,47,44,49,0]; *Cx. pipiens* s.l.*:* [212,0,218,219,205]; *Cx. salinarius:* [25,26,0,26,25]; *Ps. columbiae:* [66,59,67,0, 64]; *Ps. cyanescens:* [0,55,56,54,56]; *Ps. ferox:* [40,31,41,34,0].

**Figure 4:**
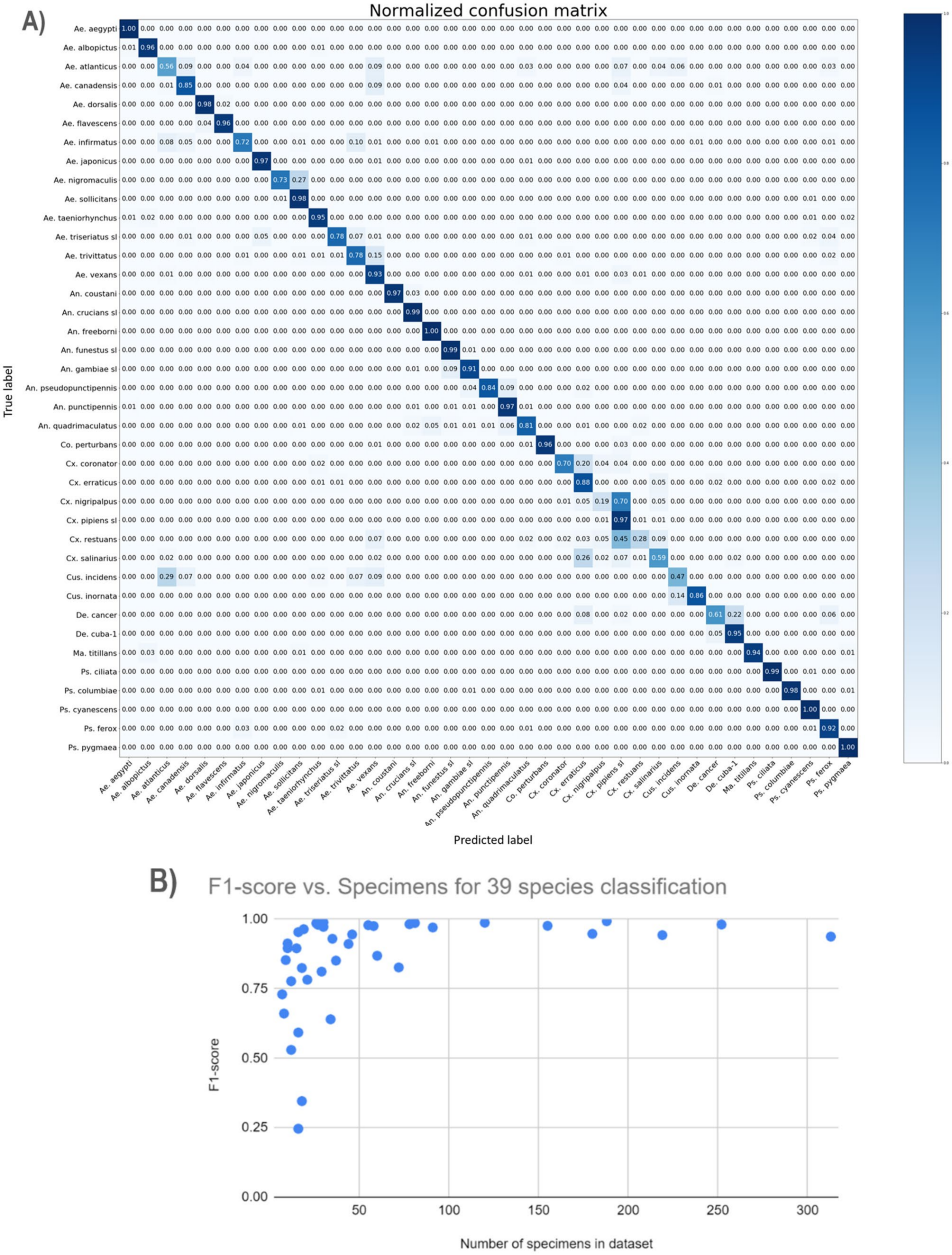
Species classification across 39 species shows the strength of CNNs for generalized mosquito classification, and elucidates a guideline for the number of specimens required for confident classification. Classification achieved unweighted accuracy of 93.06 ± 0.50% and a macro F1-score of 85.07 ± 1.81%, trained, validated, and tested over an average of 9080, 1945, and 1945 samples over five folds. (**A**) The majority of the error in this confusion matrix shows confusion between species of the same genera. Some of the confusion outside of genera is more intuitive from an entomologist perspective, such as the 10.2% of *Deinocerites cancer* samples classified as *Culex* spp. Other errors are less intuitive, such as the 28.61% of *Culiseta incidens* samples classified as *Aedes atlanticus*. (**B**) This plot of average F1-score of a species against the number of specimens which made up the samples available for training and testing shows the relationship between the available data for a given specimen and classification accuracy. When following the database development methods described in this work, a general guideline of 100 specimens’ worth of data can be extrapolated as a requirement for confident mosquito species classification.

Many of the species which were a part of the unknown datasets had enough data to perform preliminary classification experiments. Thirty-nine of the 67 species had more than 40 image samples. Species classification on these 39 species yielded an unweighted accuracy of 93.06 ± 0.50% and a macro F1-score of 85.07 ± 1.81% (see Fig. 4A). The average F1-score for any one species was plotted against the number of specimens representing the samples in the species, which elucidates the relationship between the training data available and the accuracy (see Fig. 4B). No species with more than 100 specimens produced an F1-score below 93%.

Test set sample size for each species in the 39 species closed-set classification were as follows (formatted as species, [fold1,fold2,fold3,fold4, fold5]): *Ae. aegypti:* [131,127,127,124,133]; *Ae. albopictus*: [99,99,107,97,95]; *Ae. atlanticus*: [15,13,14,14,15]; *Ae. canadensis*: [17,21,21,21,20]; *Ae. dorsalis:* [42,41,43,40,43]; *Ae. flavescens*: [13,14,14,14,14]; *Ae. infirmatus*: [17,15,19,18,16]; *Ae. japonicus*: [155,153,151,160,150]; *Ae. nigromaculis*: [6,6,5,5,5]; *Ae. sollicitans*: [63,61,58,57,60]; *Ae. taeniorhynchus*: [30,25,27,25,25]; *Ae. triseriatus* s.l.: [14,16,17,14,13]; *Ae.* *trivittatus*: [28,24,25,24,23]; *Ae. vexans*: [46,58,57,51,50]; *An. coustani*: [25,32,27,33,27]; *An. crucians* s.l.: [64,57,60,59,62]; *An. freeborni *s.l.: [85,77,82,74,89]; *An. funestus* s.l.: [181,187,166,175,161]; *An. gambiae* s.l.: [191,182,178,185,194]; *An. pseudopunctipennis*: [10,8,12,9,9]; *An. punctipennis*: [32,28,38,32,32]; *An. quadrimaculatus*: [30,33,26,37,35]; *Coquillettidia perturbans*: [31,29,30,32,35]; *Cx. coronator*: [10,9,10,11,10]; *Cx. erraticus*: [48,51,49,53,50]; *Cx. nigripalpus*: [14,14,13,13,13]; *Cx. pipiens *s.l.: [205,203,216,208,216]; *Cx. restuans*: [12,13,12,14,12]; *Cx. salinarius*: [24,25,24,23,24]; *Cus. incidens*: [9,9,9,9,8]; *Cus. inornata*: [9,9,8,9,9]; *Deinocerites cancer*: [10,10,10,10,9]; *De. sp. Cuba-1*: [16,14,15,14,15]; *Mansonia titillans*: [15,16,15,14,13]; *Ps. ciliata*: [29,26,24,23,28]; *Ps. columbiae*: [62,59,63,60,61]; *Ps. cyanescens*: [55,54,57,55,55]; *Ps. ferox*: [32,48,31,36,34]; *Ps. pygmaea*: [24,25,25,24,25].

### Comparison to alternative methods

Some intuitive simplifications of our methods, along with some common direct methods for novel species detection, are compared to our full methods. All compared methods were found to be statistically different from the full methods using McNemar’s test. The compared methods tested, along with their macro F1-score, standard deviation, and p-value as compared to the full methods, were as follows: (1) soft voting of all Tier II component outputs, without a GMM arbiter (86.87 ± 3.11%, *p* < *0.00001*); (2) the Random Forest Tier II component, appended to the closed set classification CNN from Tier I (82.80 ± 3.84%, *p* < *0.00001*); (3) the SVM Tier II component, appended to the closed set classification CNN from Tier I (82.68 ± 4.51%*, p* < *0.00001*); (4) the WDNN Tier II component, appended to the closed set classification CNN from Tier I (81.87 ± 4.53%, *p* < *0.00001*); (5) the softmax of the closed-set Xception logits, producing an unknown prediction for those specimens where no probability exceeded a threshold determined during training (72.38 ± 4.43%*, p* < *0.00001*); (6) using the predicted class of the open-set Xception model, but remapping any genus level class and the general mosquito class to the unknown class (72.72 ± 4.28%*, p* < *0.00001*); (7) ODIN paired with the closed-set Xception to recognize out of distribution classifications (49.58 ± 26.02%*, p* < *0.00001)*. Our full novelty detection methods are significantly different from each alternative method tested on these twenty-five folds, but with macro F1-score and standard deviation (86.24 ± 2.48%) similar to the simplified methods. The difference is noticeable in a higher macro-averaged unknown sensitivity for the full methods, seen in Table 3, the advantages of which are discussed in the discussion.

**Table 3:** Comparison of our methodology to simpler alternative methods.

	McNemar’s Test, *χ*^2^_*1*_	Macro F1-score	Macro unknown recall/sensitivity	Macro unknown precision
Full methods		86.24 ± 2.48%	94.09 ± 2.42%	79.66 ± 3.24%
Soft voting of all T2 components	*χ*^2^_*1*_ = *332* ^a^ *p* < *0.00001*	86.87 ± 3.11%	88.55 ± 3.58%	85.36 ± 3.93%
T2–closed-set Random Forest	*χ*^2^_*1*_ = *920* *p* < *0.00001*	82.80 ± 3.84%	83.79 ± 4.86%	81.97 ± 4.23%
T2 – closed-set SVM	*χ*^2^_*1*_ = *1120* *p* < *0.00001*	82.68 ± 4.51%	82.93 ± 5.68%	82.58 ± 4.60%
T2–closed-set WDNN	*χ*^2^_*1*_ = *905* *p* < *0.00001*	81.87 ± 4.53%	87.34 ± 5.96%	77.11 ± 3.93%
Softmax with a threshold	*χ*^2^_*1*_ = *12,151* *p* < *0.00001*	72.38 ± 4.43%	61.81 ± 4.31%	87.43 ± 5.47%
Open-set re-mapped	*χ*^2^_*1*_ = *24,656* *p* < *0.00001*	72.72 ± 4.28%	58.03 ± 5.52%	98.02 ± 1.98%
ODIN	*χ*^2^_*1*_ = *6414* *p* < *0.00001*	49.58 ± 26.02%	68.87 ± 42.70%	82.02 ± 5.77%

^a^A high chi-squared (χ^2^_1_) value dictates a low p-value, which indicates a statistically significant difference with the full methods.

## Discussion

The novelty detection algorithm presented achieves an accuracy of 89.50 ± 5.63% in identifying unknown species using field mosquito specimens, outperforming other common methods in terms of unknown sensitivity and F1-score. Cascaded with closed-set classification, the overall classification accuracy is 89.07 ± 5.58%. Closed-set classification on 16 known species achieves 97.04 ± 0.87% accuracy and a macro F1-score of 96.64 ± 0.96%. A widened closed set of 39 species classification achieves 93.06 ± 0.50% accuracy and a macro F1-score of 85.07 ± 1.81%. This work represents two landmarks in the field of computer vision-based mosquito recognition: (1) the use of wild caught specimens with highly variable physical condition for classification across a wide set of species, including morphologically similar species; and (2) accurate detection of species novel to the classification algorithm, such that practical deployment need not be limited to regions where all mosquito species are included in the database. The high accuracy species classification paired with novel species detection validates the practical use of computer vision for mosquito identification and demonstrates its potential as a reliable identification method even between species of similar morphology. Most important, the introduction of a novelty detection algorithm tested on species of similar morphology to those in the known species distribution offers a practical path to deployment of these algorithms in the field in the face of otherwise impractical database requirements, to supplement and fill gaps in current vector surveillance programs globally. In short, we have demonstrated a viable computer vision solution to the noisy, fine-grained, open-set classification problem of mosquito species identification.

A major roadblock to developing a field ready machine learning-based mosquito identification algorithm is amassing a database sufficient to recognize any one of the over 3500 mosquito species. This is difficult not only due to the distribution of these species around the world, but also the difficulty of capturing many of these species, and of taxonomists' ability to recognize them, as most taxonomists only learn the species of a region relevant to their work. By introducing the unknown category, we can focus on training algorithms to identify common and medically relevant species, with confidence that species outside of our dataset will be categorized as an unknown species. Furthermore, classification with 39 species of limited data shows that species with more than 100 specimens produce confident classifications above 90%. Some reduction in classification accuracy may be due to extreme class imbalance in this case despite implementing focal loss to control for this. Regardless, though some species may be more distinct than others, enabling high accuracy with smaller datasets, 100 specimens may be used as a guideline for data requirements for mosquito species classification with computer vision, until more thorough research on this question has been done. This work defines and dramatically reduces the data necessary for mosquito classification with computer vision as a useable tool, clarifying a viable path forward for practical use by vector control organizations for disease prevention.

Although the overall accuracy of the 16 species classification is high (97.04 ± 0.87%), performance was markedly lower for certain species. However, 73.5% of the error occurred within the genus of the sample. Though the error for identifying *Culex salinarius* was high, with 20.6% of samples confused with *Culex erraticus* and 6.1% confused with *Culex pipiens sl*, similar error within a genus is also more common among human experts, particularly within *Culex*. The authors attribute the error which lies outside genera to three compounding factors: (1) the limited amount of data available, given that 97% of the species normalized error that occurred outside of genus involved species with fewer than 100 specimens; (2) the stochastic nature of training deep neural networks, which in the face of limited data may produce unexpected error distribution; (3) and the highly damaged nature of many of the specimens in the image database produced for this work, which may also be minimized by increasing training dataset size. Future work should investigate novelty detection as a broader application of distinguishing both novel species and low confidence specimens from closed-set classifications. Furthermore, other variables could be distinguished alongside species identification, such as mosquito sex, age, and feeding state. Analysis of these attributes may impact species identification accuracy, as well as provide additional value to mosquito control organizations. Given the diversity of species in different regions, inclusion of geographical and temporal information for time and place of capture may improve classification accuracy, as was recently done for butterfly classification^28^, another fine grained insect classification problem.

The novel, or unknown, species detection method in this work is important both to mosquito identification and to the field of computer vision. Previous work in recognizing novel classes, such as Outlier detection in neural networks (ODIN) and others described in the review by Geng et al. on open set recognition methods, has been focused on novel classes far outside the distribution of known classes^26^. However, these algorithms do not perform well on extremely fine-grained tasks, such as that presented in this work. Closed-set computer vision algorithms, such as those presented in previous works on mosquito classification, can only classify species on which they have been trained. This is similar to current entomological practice which relies on dichotomous keys that typically only contain a select group of species common to a particular region. If a mosquito belongs to a species not in the key, a technician will either mistakenly label it as a species they know, or they will recognize the anomaly and set the specimen aside for further analysis. Accurately identifying unknown species is a challenging problem because there is no visual or morphological reason for the distinction; it is an artificial product of what the user, or algorithm, is familiar with. Since a species’ unknown status is not necessarily indicative of visual characteristics far distinguished from the known species, the differences may not be an abstraction of the images themselves, but an abstraction of the features generated by a CNN processing the image in attempting species classification. This theory is the basis for the tiered approach of the novelty detection algorithm as presented in this work, where the features are analyzed by tiers of classifiers which are trained after the initial training of the CNNs. Disjointing the learning of feature extraction from the novelty detection portions of the methods and isolating feature extraction learning to species classification enables the later tiers to be independent arbiters of the novelty of the species in question. Future work should attempt to improve upon these methods, testing them on similar many-class fine-grained classification problems with inherent class imbalance issues.

The markedly lower performance of N set as compared to U1 set and U2 set is expected given that the CNNs did not train on the N set species. The difference between the micro-averaged and macro-averaged accuracies of N set (80.83 ± 19.91% and 88.72 ± 5.42%) indicates that the species within N set which contain the most specimens also tend to be the species most distinct from the K set, despite species mix-up with cross-validation. However, it is notable that the U2 set species achieved high micro-averaged and macro-averaged accuracies (97.05 ± 1.94% and 97.30 ± 1.41%). Many of the U2 species contained only a few samples. This suggests that if only a few specimens are obtained from otherwise unknown species, the species can be included in U2, and thereafter provide little threat of error regarding our unknown algorithms. The novelty detection accuracy for the K set known species was lowest of the species sets (80.79 ± 7.32% micro-averaged and 75.83 ± 4.65% macro-averaged). Though some of the compared novelty detection methods exceeded the full methods in known recall, or known sensitivity, the full methods are preferable from an entomologist perspective because they bias answers towards unknown. Mosquito taxonomists are conventionally trained to only provide confident answers. For example, if they think a specimen in question may be *Culex erraticus* but they are unsure, they are encouraged to call the specimen *Culex spp.* if they are confident of the genus*,* or to family Culicidae if they are not. In this way, the full methods, and in particular the inclusion of the GMM arbiter in Tier III, are preferable to the simpler alternatives, as they bias answers towards unknown, rather than known.

A potential application of this technology is in the recognition of malaria vectors. A major challenge to malaria elimination is weak surveillance, monitoring, and evaluation^29^. Although long-lasting insecticide nets and indoor residual spraying efforts have yielded progress, malaria elimination has been set back in many countries by insecticide resistance, the misuse of interventions, host behavior (such as cooking outdoors in the early evening) and adaptive vector behavior (such as biting earlier in the evening, before humans are under the protective cover of bed nets). In sub-Saharan Africa, the primary malaria vectors are *Anopheles gambiae* s.l. and *Anopheles funestus* s.l., species complexes that are often difficult to distinguish from each other and other *Anopheles* species. Ideally, public health systems should know the density, composition, and distribution of the mosquito species in a finite region, allowing them to optimize interventions to a particular area^2^. If an affordable computer vision system were to be deployed in the field, organizations without access to specially trained taxonomists could obtain these composition and distribution patterns in a more standardized and timely manner.

The implications of this work for the prevention of mosquito borne diseases are vast. The first step towards implementing an effective control program is obtaining high quality data on the disease vectors in an area^3,30^. Without robust vector surveillance, emerging vector-borne disease threats cannot be addressed. Current global vector surveillance systems are unstandardized, burdensome on public health systems, and threatened by the global paucity of entomologists. Such a computer vision system could empower non-experts to accurately and rapidly identify disease vectors in the field, increasing programmatic capability and capacity with minimal training and cost.

## Methods

### Image database development

A diverse database of mosquito images was developed primarily from desiccated mosquito specimens from field sites across Africa and North America, but was supplemented by lab colony specimens from species for which field specimens were difficult to obtain. Specimens ranged in condition from fully intact to missing appendages with badly damaged head and/or body (see Supplementary Fig. 1, Image Database Preview). Each mosquito was identified by either genetic identification or human expert identification under a microscope. Expert identification was only used when genetic identification was unavailable, or the mosquito was grown in a lab colony. The molecular identification approach used was DNA-barcoding performed by Walter Reed Biosystematics Unit (WRBU), where DNA signatures are compared against the gold-standard Mosquito Barcoding Initiative reference libraries^31–33^, or identified using specific nuclear DNA ITS2 assays where available^34,35^. All DNA barcodes generated for mosquitoes imaged in this study are publicly available through GenBank. Associated data and raw chromatograms are hosted on the Barcode of Life Database (BOLD; www.boldsystems.org), under the container JHUSM and project codes SMARU, SMARV, SMARW and SMARX. Each mosquito was imaged at multiple random orientations on a white background, resulting in 4–10 images per mosquito. By this process, 12,977 photos of 2,696 specimens from 67 species were generated, (see Supplementary Table 2, Image Database Details). Of the 2696 specimens, 53.1% were identified using DNA and 86.4% were wild caught, and of the 67 species, 95.5% consisted solely of wild-caught specimens. The number of images for each species were as follows: *Ae. aegypti* 921, *Ae. albopictus* 692, *Ae. atlanticus* 80, *Ae. canadensis* 128, *Ae. cantator* 8, *Ae. condolescens* 10, *Ae. dorsalis* 288, *Ae. fairfax-1* 6, *Ae. flavescens* 72, *Ae. hendersoni* 16, *Ae. infirmatus* 106, *Ae. japonicus* 1093, *Ae. mediovittatus* 84, *Ae. melanimon* 20, *Ae. nigromaculis* 42, *Ae. sierrensis* 8, *Ae. sollicitans* 414, *Ae. spilotus* 4, *Ae. sticticus* 4, *Ae. taeniorhynchus* 184, *Ae. tortilis* 21, *Ae. triseriatus s.l.* 87, *Ae. trivittatus* 143, *Ae. vexans* 364, *An. cf-coustani* 21, *An. coustani* 142, *An. crucians s.l.* 278, *An. freeborni* 570, *An. funestus s.l.* 1210, *An. gambiae s.l.* 1160, *An. maculipalpis* 30, *An. pharoensis* 6, *An. pretoriensis* 12, *An. pseudopunctipennis* 53, *An. punctipennis* 212, *An. quadrimaculatus* 195, *An. rufipes* 12, *An. squamosus* 13, *An. tenebrosus* 6, *An. ziemanni* 35, *Co. perturbans* 204, *Cx. antillummagnorum* 28, *Cx. bahamensis* 4, *Cx. coronator* 61, *Cx. erraticus* 343, *Cx. nigripalpus* 74, *Cx. pipiens s.l.* 1503, *Cx. restuans* 80, *Cx. salinarius* 163, *Cx. tarsalis* 14, *Cx. territans* 13, *Cu. incidens* 45, *Cu. inornata* 46, *Cu. melanura* 5, *De. cancer* 39, *De. cuba-1* 72, *Mansonia titillans* 93, *Orthopodomyia signifera* 18, *Ps. ciliata* 169, *Ps. columbiae* 423, *Ps. cyanescens* 390, *Ps. discolor* 10, *Ps. ferox* 231, *Ps. howardii* 17, *Ps. pygmaea* 149, *Ps. signipennis* 23, *Uranotaenia sapphirina* 10.

Specimens were photographed with a handheld, USB microscope (Zarbeco MiScope Handheld Digital Microscope—MiSC-s2) set at 40X zoom, producing images of 640 × 480 pixels. The resolution of the microscope at 40X zoom was measured at 28 line pairs/millimeter, or 18 resolvable microns, characterized using the United States Air Force 1951 Resolution Chart. During training, images were made square by adding a patch of background color to the top and bottom of each image, by taking the mean of each channel for the top 5 and bottom 5 rows of the image. After being made square, the images were down sampled to 299 × 299 pixels before being fed to the CNNs to match the image size of the selected architecture. Of the 67 species, 20 species were selected to be considered known based on the number of specimens in the database, the species’ medical relevance, and addressing class balance between known and unknown. For each of the five data folds, 4 of the 20 known species were removed to the unknown category, leaving 16 known species for each fold. Each species was considered unknown for one of the five folds. To maximize the potential of the limited dataset, we used real time data augmentation, using the default parameters of the get_transforms function in the FastAI PyTorch library^36^ (random cropping, affine transform, symmetric warp, rotation, zoom, brightness, and contrast modifications with the exception of the affine transform, for which a dihedral affine was used).

### Novelty detection algorithm structure

The three tiers of the novelty detection algorithm are trained independently in sequence. Different data is used to train Tier I than is used to train Tiers II and III, so that Tiers II and III can assess the familiarity of Tier I CNNs with the new data presented it, rather than training the later tiers on data to which Tier I was fit during its training.

Tier I uses two CNNs trained independently on the same data. The models trained were both Xception^37^ models, but were trained on different data with different class divisions. The Xception model uses separable convolutions which reduces the number of parameters in the model. This in turn reduces the network's tendency to overfit, while also reducing training and inference time. These hypotheses were further verified through experimental validation by comparing to other standard CNN architectures. The two datasets were open-set and closed-set, where the closed-set model contained only 16 known species-level classes, and the open set model contained the 16 known species-level classes as well as 4 genus-level classes and a general mosquito class. The genus-level classes are simply formed of groups of species of the same genus and do not include any specimens of the 16 known species. The mosquito class consists of multiple genera outside the genera described by the other 20 classes. The CNNs were trained with a fully connected classification head, the open-set Xception with 21 nodes and the closed-set Xception with 16 nodes. For Tier I, in the training of Xception^37^ models we use a learning rate of 1e−2, with one cycle schedule^38^. Models are trained for 60 epochs with Ranger optimizer^39^ with a batch size of 16. We use categorical Focal Loss^40^ to address class imbalance in our data classes, with the gamma parameter set to 2. Focal loss dynamically adjusts the class weights giving hard examples higher weight, which enables better training of the model in the face of imbalanced classes. Model training is performed on an RTX 2070 Super GPUs using the deep learning framework PyTorch^41^.

Tier II consists of three standard machine learning classifiers, a support vector machine (SVM), a random forest (RF), and a wide and deep neural network (WDNN)^42^, appended to each the Tier I CNNs, resulting in six Tier II components. Tier II components were selected through preliminary iterative experimentation with various models of similar complexity, evaluating their capability of parsing through the features and probabilities produced by Tier I. Each attempted to determine whether the given specimen was from a known species or an unknown species by analyzing the features of the Tier I CNN, and for the WDNN, both the features and the logits. The SVM uses a Radial-basis function (RBF) kernel, with a kernel coefficient (γ) set according to γ = *1/*(*n*_*features*_ * X_*var*_), where *n*_*features*_ is the number of features per sample and the *X*_*var*_ is the variance of all samples in the set^43^. It derives a squared L2 penalty based on a regularization constant (C) set to 0.1. The RF fits 200 decision trees with a maximum depth of 19, considering log_2_(*n*_*features*_) features per split and using entropy as a measure of impurity^43^. The WDNN is a deep neural network with two input layers, a deep input which learns lower dimensional dense representations of the data and a wide input which memorizes shallow features to help protect the network from over-generalization. The deep input takes the features (2048 nodes) and passes them to four hidden layers (2500, 1900, 1900, and 1900 nodes). It uses He normal initialization^44^, and includes a 0.65 dropout layer^45^, a batch normalization layer^46^, and an ELU activation^47^ between each hidden layer. The wide input receives the logits (21 for open-set Xception and 16 for closed-set Xception) and directly concatenates them with the final hidden layer of the deep network. This concatenated layer feeds to a single output node to determine known or unknown. The network is trained for 20 epochs using a binary cross entropy loss function with an Adam optimizer^48^. The learning rate was initially set to 1e-3, decaying every 10 epochs at a rate of 0.1.

Tier III first intakes the unknown probabilities from the six Tier II components and uses soft voting for the initial assessment (i.e. averaging the six probabilities). Average probabilities, *p*_*avg*_, are then sorted into confident and unconfident predictions, based on thresholds determined during training. Unconfident predictions are those where *T*_*known*_ < *p*_*avg*_ < *T*_*unknown*_, where *T*_*unknown*_ = 0.9, above which an unknown prediction is considered confident, and where *T*_*known*_ = 0.3, below which a known prediction is considered confident. Unconfident predictions, where the average probability falls between the two thresholds, are concluded by evaluating the six tier II probability scores, *p*, with a Gaussian Mixture Model (GMM) in sckitlearn^43^. A GMM was selected because it facilitated moving uncertain *p* predictions to unknown as much as possible, minimizing erroneous known predictions. The GMM performs unsupervised clustering of known samples, treating unknown samples as anomalies. It fits the probabilities from the six Tier II components into seven clusters, using a random 10% of the unknown training data for Tier II/III and all of the known training data. We estimate the density of the model for each sample using the score_samples method, which finds the log of the probability density function at that location. The lower the score, the lower the density and the less likely that the given sample is in the known set. This density is used to measure the likelihood that a datapoint fits into any of the seven clusters in the model, and the lowest 10% of these are considered anomalous, or in this case unknown. The 10th percentile defines a density threshold, below which a sample is determined to be unknown.

Hyper parameter optimization for Tier II and III components was conducted on the training data for seed 0 of fold 1 using a random search. The rate limiting factor for training time is the CNN Xception, which contains 22,855,952 parameters. Even so, the training of the closed-set Xception using our methods and data schema took on average 87 min for a single fold. The RF, SVM, WDNN, and GMM each respectively take (3.4 s (s), 9.8 s), (13.1 s, 12.6 s), (25.7 s, 20.4 s), and 2.7 s to train for a single fold on a CPU, formatted (closed, open) for Tier II components. The short training time is due in large part to the small size of the dataset.

### Data partitioning and training

Training of the novelty detection algorithm to recognize when a specimen’s species is known or unknown to the closed-set CNN from Tier I results in three distinct types of data to be encountered in the wild: specimens from known species recognized by the closed-set CNN (K); specimens from unknown species used to train the algorithm (U); and specimens from novel unknown species which were not used in training (N). We evaluate the algorithm on representations of each of these populations, which we refer to as species sets K, U, and N.

Sixty-seven mosquito species were included in the overall dataset. Twenty of these species were included in the K dataset. All other species were always used in either the U or N datasets. Each specimen had multiple photos, and each photo was considered a single sample. When a specimen was designated for training, validation, or testing for a particular fold, all photos of that specimen were isolated to that activity.

Tier I closed-set CNNs were trained only on K species. Tier I open-set CNNs were trained on K species and a subset of U species (U1). The Tier II and III classifiers were trained on K species, U1 species, and the remaining subset of U species (U2). The remaining set of unknown species not used in training comprised the novel species testing set N. These species sets are further described as those used as inputs to Tiers I, II, and III (population represented by group U1), those used as inputs to train Tiers II and III (population represented by group U2), and those species which are novel to the algorithm (population represented by group N).

The algorithm training and evaluation was iterated on multiple data splits, assessing different divisions of species across sets K, U1, U2, and N. In each of the five folds, 4 of the 20 species considered known were removed from the K set for cross validation, leaving 16 species within the K set for each fold. U1 species set, a subset of 18 unknown species for training the open-set Tier I CNN, also trained on by the Tier II and III components, remained the same species for all folds and iterations, throughout training and testing. The remaining unknown species were distributed between U2 and N sets. Tier I was iterated on five distinct folds with data from sets K and U1 using a training:validation:testing split ratio of 70:15:15.

Tier II and III components were trained using the testing data of Tier I. The data split was iterated 5 times for each of these five folds with data from sets K, U1 and U2, resulting in twenty-five iterations. For each fold and iteration, the specimens within each set designated for training and testing were randomized. For each iteration, the unknown species which were designated for N versus U2 were iterated, being sure that each species was included in the N set for at least one iteration, except U1 species which could not be iterated in this way. For each iteration, the percentage of specimens within each species to be designated for training or testing was first set by a training:testing split ratio of 80:20, then manipulated to reduce class imbalance between known and unknown within a genus and between morphologically similar species (see Supplementary Table 1, Datafolds).

### Species classification

After the novelty detection methods, if a sample was considered known, the sample was sent to be classified by the Tier I closed-set CNN. The results are first calculated independent of the novelty detection methods, including all samples of the species in question which were not included in training or validation for the training of that fold. Because each fold removed four species from the dataset, such that each species was considered unknown for one of the five folds, there was an imbalanced number of iterations distributed across the confusion matrix, thus each cell is averaged independently. The results are then calculated as a subsequent step after the novelty detection methods, only classifying species on samples first determined to be known, and where unknown is maintained as a class within the confusion matrix. This confusion matrix was calculated over the twenty-five folds of the novelty detection methods, using the respective Tier I CNN for that fold. Both confusion matrices are normalized.

Of the 67 species recorded in our dataset, 39 of them have more than 40 photos, and were thus included in the widened closed-set species classification, which was trained over five data folds on the same architecture and hyper parameters stated for training the Tier I closed-set CNN. The five data folds simply iterated the distribution of specimens across the training:validation:testing split. The average macro-F1-score and average accuracy, and their standard deviations over the five folds were reported, along with an average normalized confusion matrix, and a graph comparing the average accuracies to the number of specimens in the database.

### Analysis methodology of detecting novel species

For each of the fifty iterations, the algorithm’s performance on both known and unknown species was evaluated. The accuracy, unknown sensitivity/recall, unknown specificity, and unknown precision was calculated for each iteration, and mean and standard deviation of each species set (K, U1, U2, and N) was reported. Micro-average results are reported considering each sample with an equal weight, and macro-average results are reported considering the mean for each species with an equal weight. A mean confusion matrix was calculated using closed-set Xception model results for the five folds. The 20 known species were considered as distinct classes, and the remaining species grouped together as a distinct unknown class. Each of the twenty-five iterations of Tier II and III was cascaded within its respective fold of the five folds of Tier I to calculate the unknown detection and species classification confusion matrix.

In addition, simpler methods for novelty detection were compared to our methods to assess the need for the complexity of our novelty detection algorithm (see Table 3). The simpler methods are as follows: our full methods without the GMM arbiter for low confidence samples, such that the final prediction is made by soft voting; the Tier II RF component appended to the Tier I closed-set CNN; the Tier II WDNN component appended to the Tier I closed-set CNN; the Tier II SVM component appended to the Tier I closed-set CNN; the Tier I open-set CNN, where any of the five open-set classes are considered unknown classifications; a softmax applied to the logits of the Tier I closed-set CNN, where unknown classification is given if no species receives a probability higher than a threshold determined through evaluation on the training set (0.99); the ODIN algorithm by Liang et al.^49^, unaltered from the author provided github, trained using the closed-set Xception, using all K set data used in Tier I training and Tier II and III training for training data inside the distribution. McNemar’s Test^50^ was used to compare the accuracies between each alternative method and our methods to assess discordance. These comparisons were performed on the same data folds, so the comparison was paired. The known and unknown precision and recall were also reported for each method.

### Statistics

The accuracy metrics used throughout this work were accuracy, precision, recall (equivalent to sensitivity), specificity, and F1-score:

1 $$F1~score~ = ~2*\frac{{Precision~*~Recall}}{{Precision~ + ~Recall}}$$

Macro F1-score is determined by calculating the F1-score for each species, then averaging the F1-score across species. This controls for class imbalance in the test set. For fold 2, all samples for *An. quadrimaculatus* were used in training the known set, so for cascading the unknown detection with closed-set classification the F1-score for this species was omitted, as there was no possibility for true positives by which to calculate it. The error bars were calculated using sample standard deviation:

2 $$s_{x} = \sqrt {\frac{{\sum \left( {x_{i} ~ - ~\underset{\raise0.3em\hbox{$\smash{\scriptscriptstyle-}$}}{x} } \right)^{2} }}{{n~ - ~1}}}$$

where *s*_*x*_ is the standard deviation, *x*_*i*_ is a given sample of measurement, $\underset{\_}{x}$ is the average of the sample population in question, and *n* is the number of samples. When comparing the difference between methods, given that the testing and training data was paired through all folds and iterations, McNemar’s Test with continuity correction was used to assess significance:

3 $$\chi ^{2} _{1} = \frac{{\left( {\left| {b~ - ~c} \right| - 1} \right)^{2} }}{{b~ + ~c}}$$

where *b* is the number of samples where our method was correct and the alternative method in question was incorrect, *c* is the number of samples where the alternative method in question was correct and our method was incorrect, and χ^2^_1_ is the test statistic with a chi-squared distribution and 1 degree of freedom.

## Supplementary Information

Supplementary Information 1.

Supplementary Information 2.

Supplementary Information 3.

## Data Availability

The datasets generated during and analyzed during the current study are available from the corresponding author upon reasonable request.

## Code availability

The code used to generate the results in this study are available in the GitHub repository: https://github.com/vectech-dev/novel-species-detection.
