# Supplementary material for: Mosquito species identification using convolutional neural networks with a multitiered ensemble model for novel species detection
Source: Sci Rep. 2021 Jul 1;11:13656. doi: 10.1038/s41598-021-92891-9 (PMC8249627; doi:10.1038/s41598-021-92891-9)
Supplement: Supplementary file 3 — Supplementary Information 3. [file 41598_2021_92891_MOESM3_ESM.docx]

Mosquito species identification using convolutional neural networks with a multitiered ensemble model for novel species detection

Authors: Autumn Goodwin^1,2^ *, Sanket Padmanabhan^1,2^, Sanchit Hira^2,3^, Margaret Glancey^1,2^, Monet Slinowsky^2^, Rakhil Immidisetti^2,3^, Laura Scavo^2^, Jewell Brey^2^, Bala Murali Manoghar Sai Sudhakar^1^, Tristan Ford^1,2^, Collyn Heier^2^, Yvonne-Marie Linton^4, 5, 6^, David B. Pecor^4, 5, 6^, Laura Caicedo-Quiroga^4, 5, 6^, Soumyadipta Acharya^2^ *

Author Affiliations:

^1^ Vectech, Baltimore, MD 21211, USA

^2^ Center for Bioengineering Innovation and Design, Biomedical Engineering Department, Whiting School of Engineering, The Johns Hopkins University, Baltimore, MD 21218, USA

^3^ The Laboratory for Computational Sensing and Robotics, Whiting School of Engineering, The Johns Hopkins University, Baltimore, MD 21218, USA

^4^ Walter Reed Biosystematics Unit (WRBU), Smithsonian Institution Museum Support Center, Suitland, MD 20746, USA

^5^ Walter Reed Army Institute of Research, Silver Spring, MD 20910, USA

^6^ Department of Entomology, Smithsonian Institution-National Museum of Natural History, Washington, DC 20560, USA

* Corresponding author(s).

**Image Database Preview**


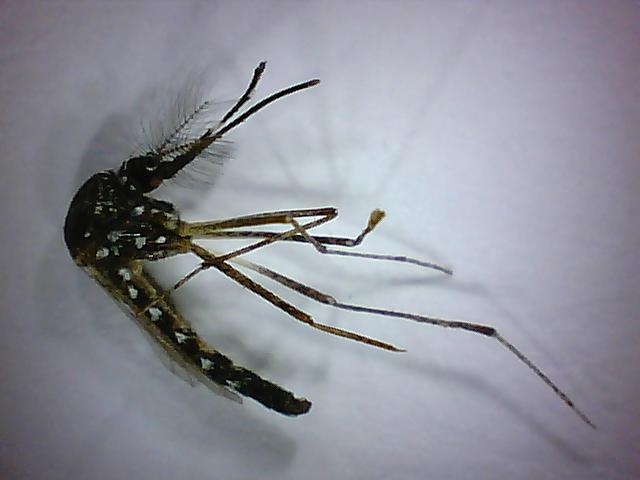

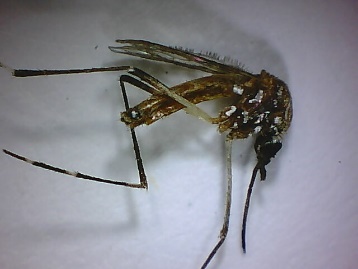

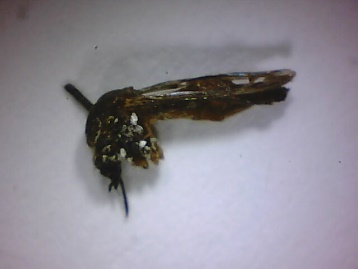

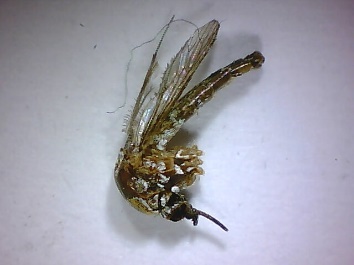


*Aedes aegypti*: JHU-000538_01m.jpg, JHU-005773_03m.jpg, JHU-005882_02m.jpg, JHU-05983_03m.jpg


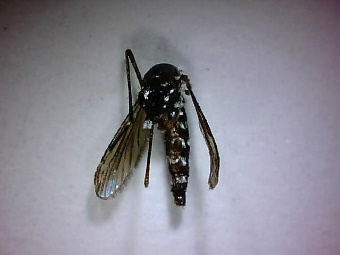

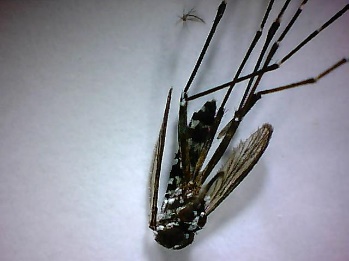

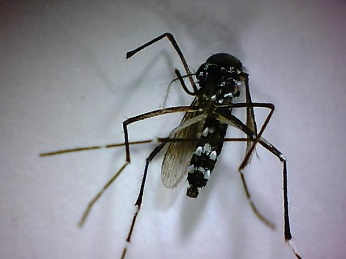

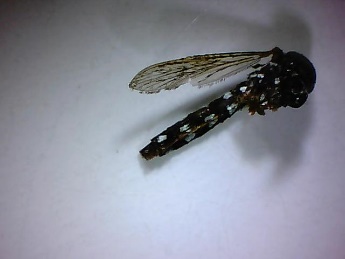


*Aedes albopictus*: JHU-001320_04m.jpg, JHU-001413_06m.jpg, JHU-001449_01m.jpg, JHU-000373_04m.jpg


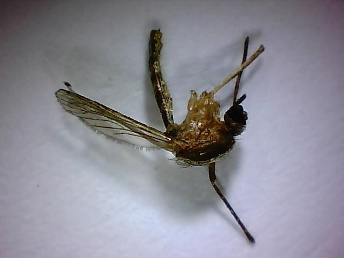

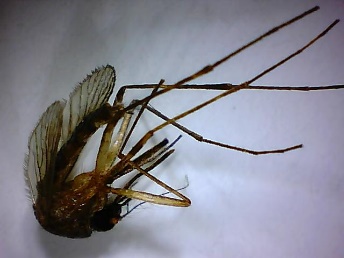

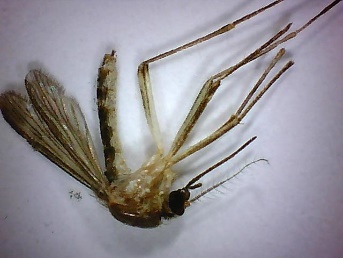


*Aedes atlanticus*: JHU-00210_01m.jpg, JHU-00519_05m.jpg, JHU-002464_04m.jpg.


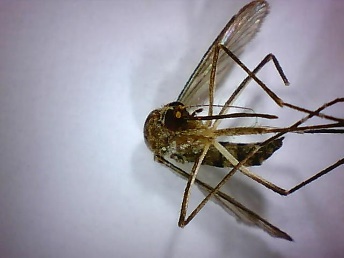

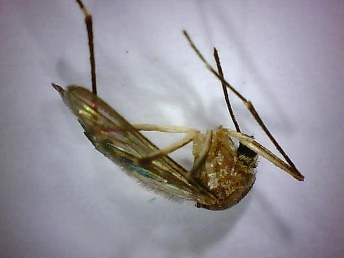

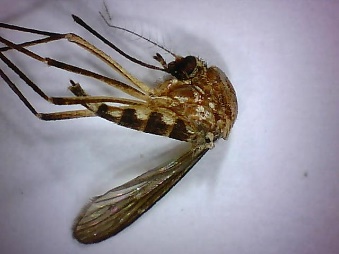


*Aedes canadensis*: JHU-005270_04m.jpg, JHU-005272_05m.jpg, JHU-005353_05m.jpg


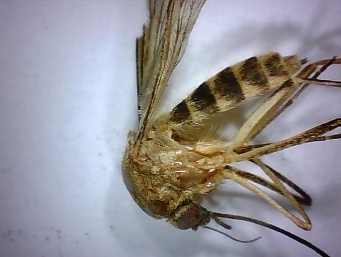


*Aedes cantator:* JHU-005331_04m.jpg


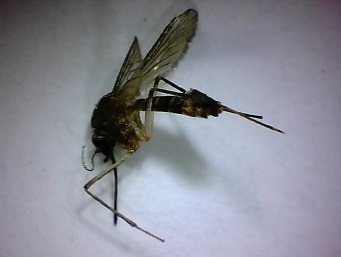


*Aedes condolescens*: JHU-000460_02m.jpg


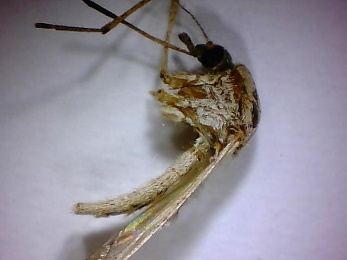

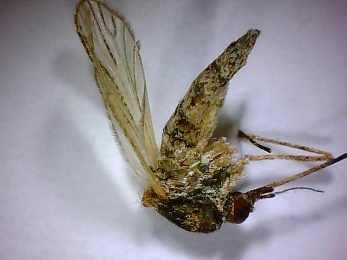

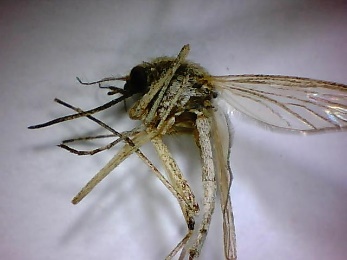

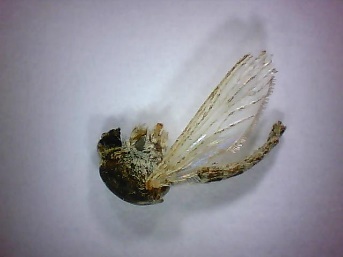


*Aedes dorsalis:* JHU-001989_04m.jpg, JHU-002093_01m.jpg, JHU-002094_06m.jpg, JHU-005277_01m.jpg


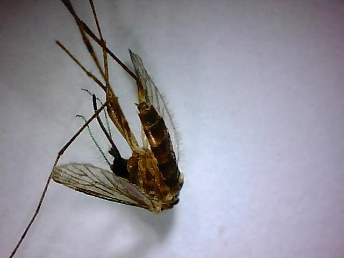


*Aedes fairfax*-1: JHU-001390_06m.jpg


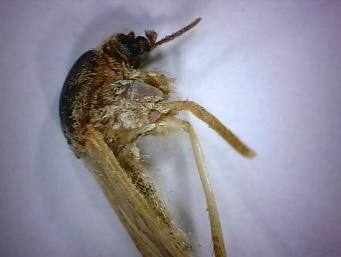

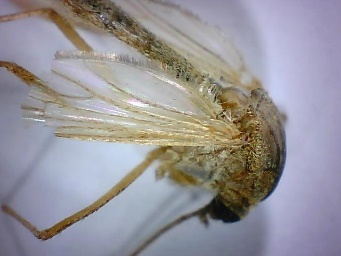

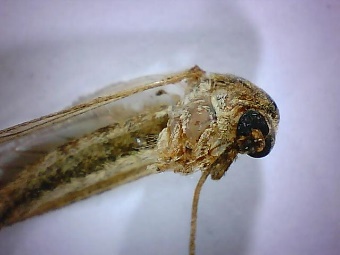

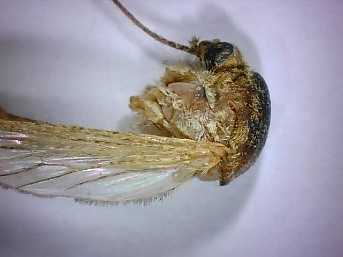


*Aedes flavescens*: JHU-005313_03m.jpg, JHU-005316_03m.jpg, JHU-005334_02m.jpg, JHU-005335_02m.jpg


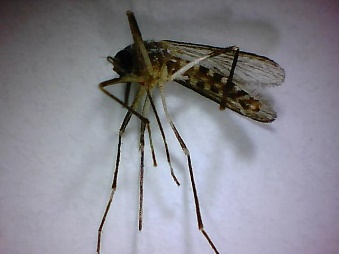

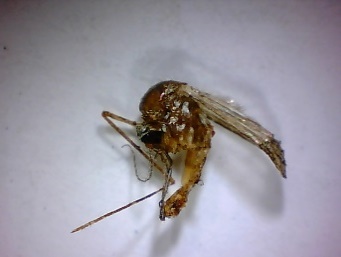


*Aedes hendersoni*: JHU-001020_02m.jpg, JHU-001176_03m.jpg


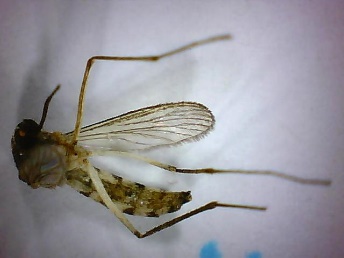

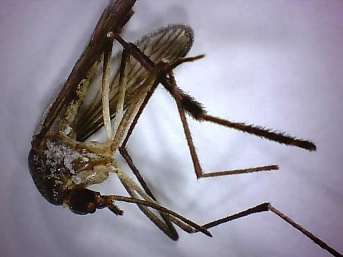

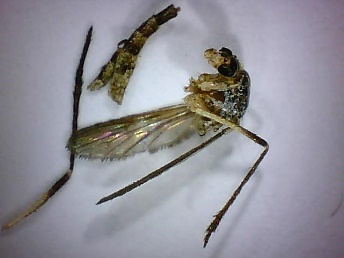


*Aedes infirmatus*: JHU-002479_01m.jpg, JHU-002489_05m.jpg, JHU-002497_01m.jpg


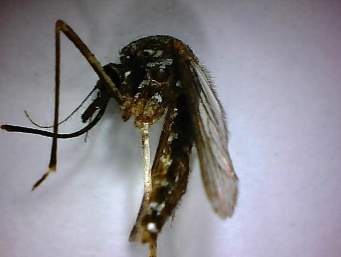

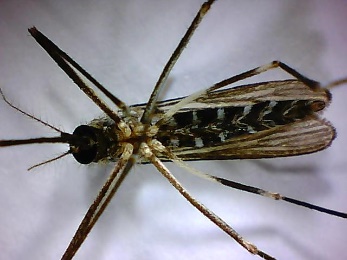

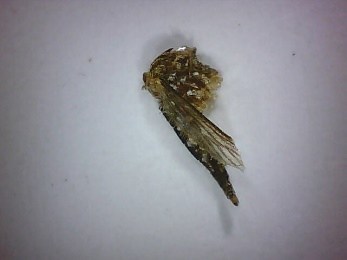

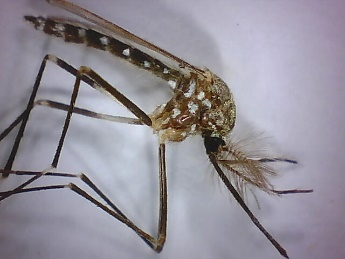


*Aedes japonicus*: JHU-001307_03m.jpg, JHU-001461_03m.jpg, JHU-007294_03m.jpg, JHU-007395_04m.jpg


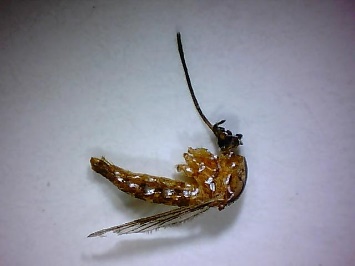


*Aedes mediovittatus*: JHU-000433_02m.jpg


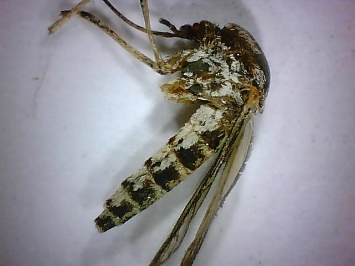


*Aedes melanomin*: JHU-002407_04m.jpg


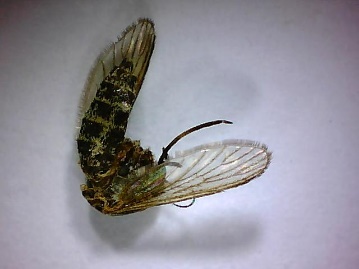


*Aedes nigroculis*: JHU-002069_01m.jpg


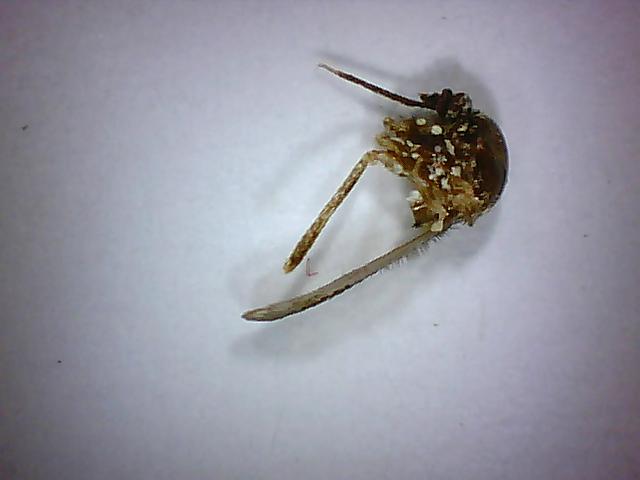


*Aedes sierrensis*: JHU-005342_08m.jpg


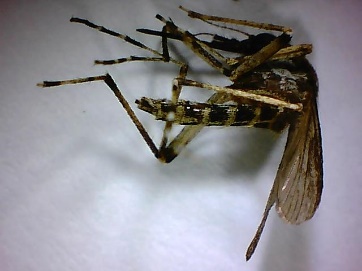

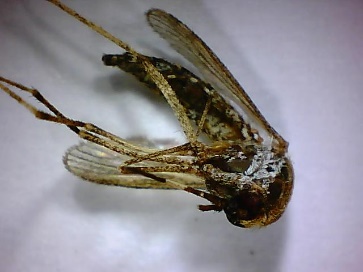

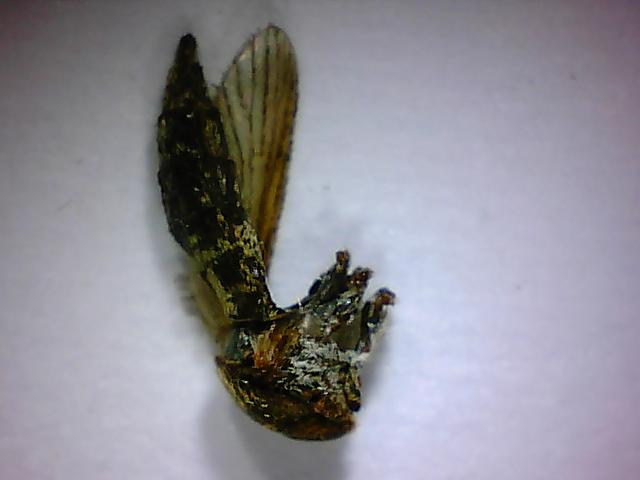


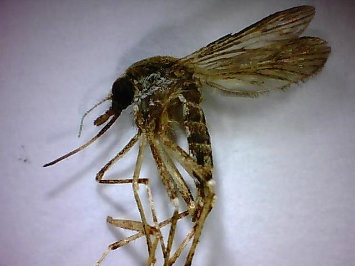


*Aedes sollicitans*: JHU-000193_01m.jpg, JHU-002011_02m.jpg, JHU-002037_02m.jpg, JHU-002054_01m.jpg


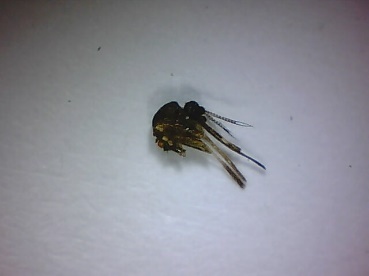


*Aedes spilotus*: JHU-05846_01m.jpg


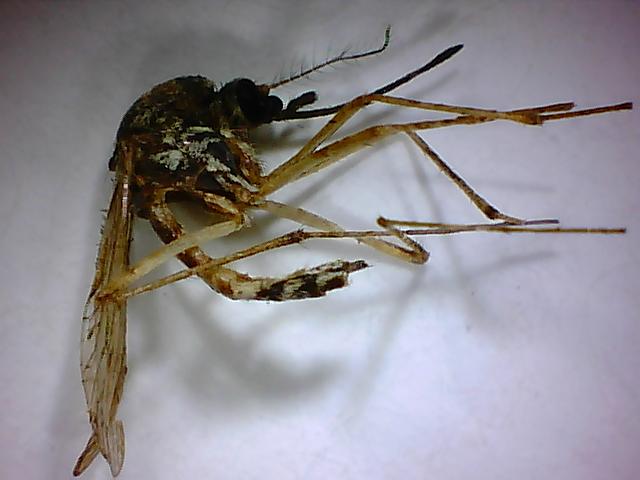


*Aedes sticticus*: JHU-001117_02m.jpg


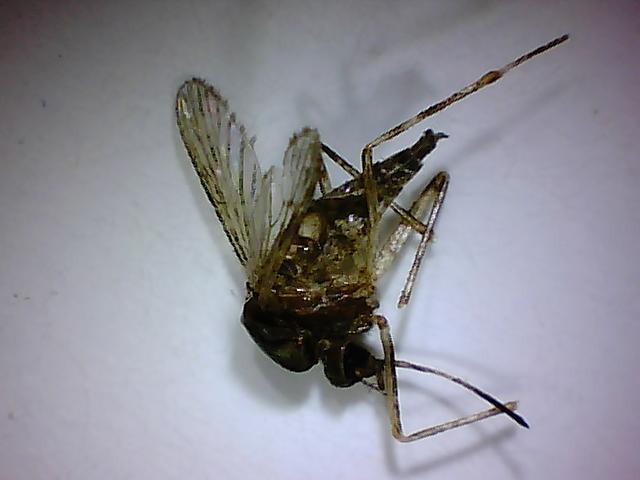

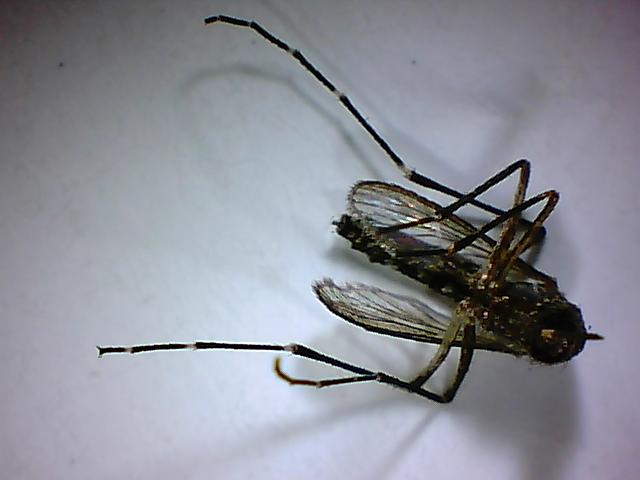

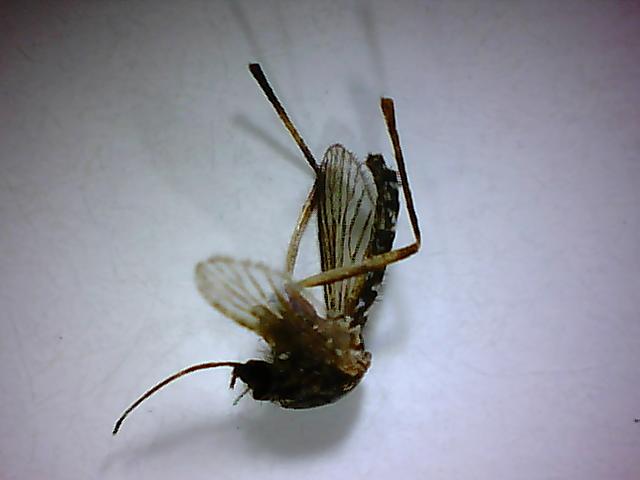

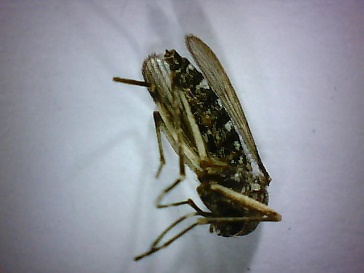


*Aedes taeniorhynchus*: JHU-000342_05m.jpg, JHU-000439_04m.jpg, JHU-000446_01m.jpg, JHU-000655_01m.jpg


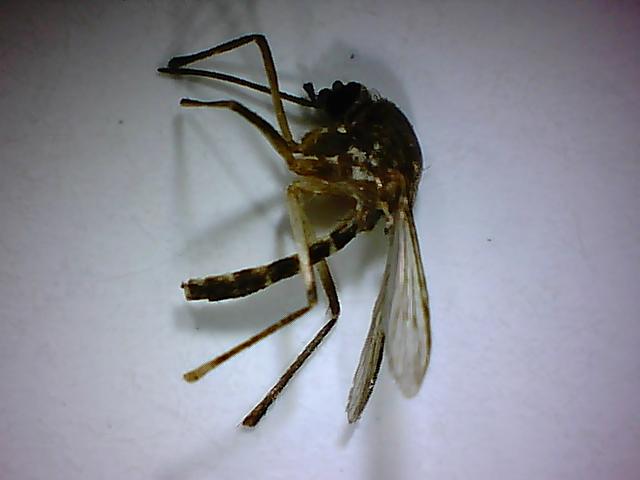


*Aedes tortilis*: JHU-000459_03.jpg


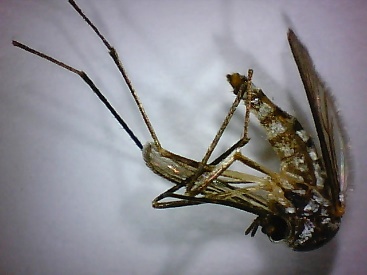

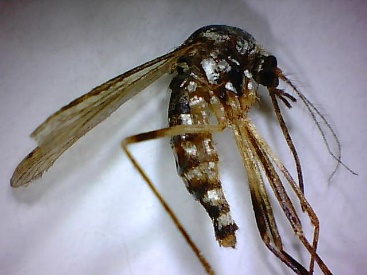

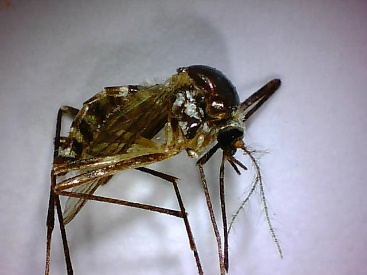


*Aedes triseriatus sl*: JHU-000643_04m.jpg, JHU-001085_05m.jpg, JHU-001289_06m.jpg


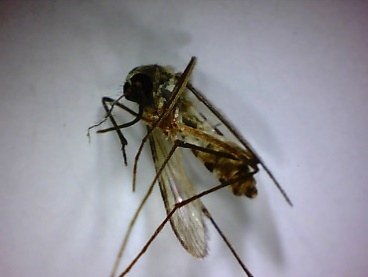

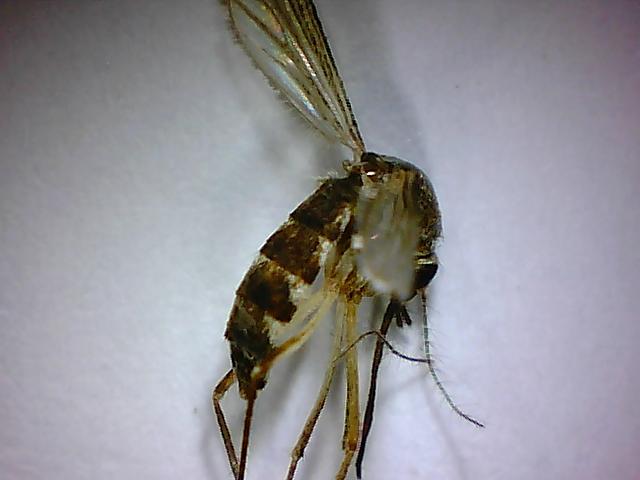

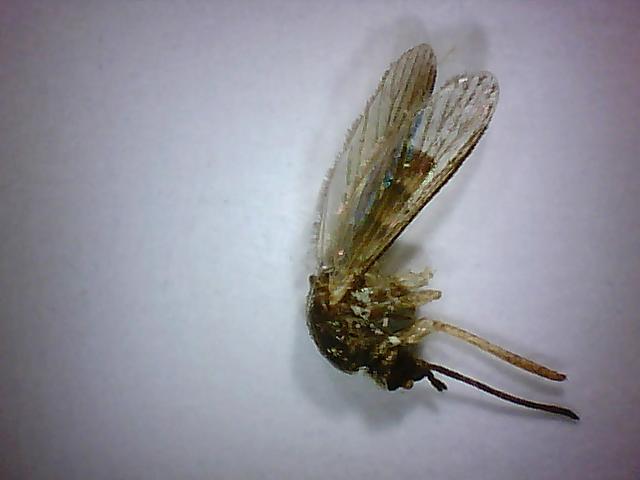


*Aedes* *trivittatus*: JHU-001250_04m.jpg, JHU-001471_04m.jpg, JHU-005293_05m.jpg


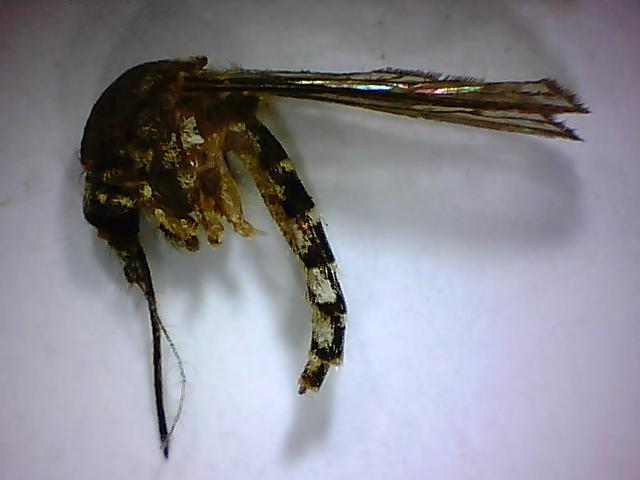

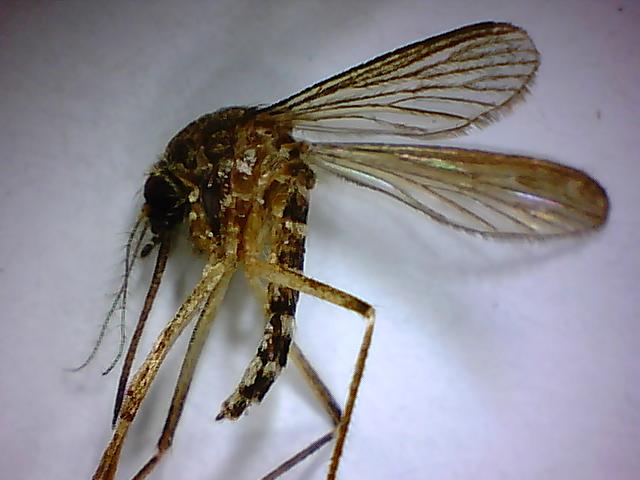

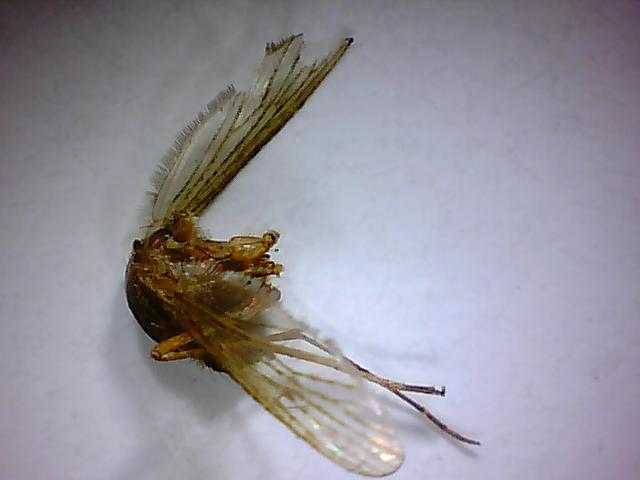

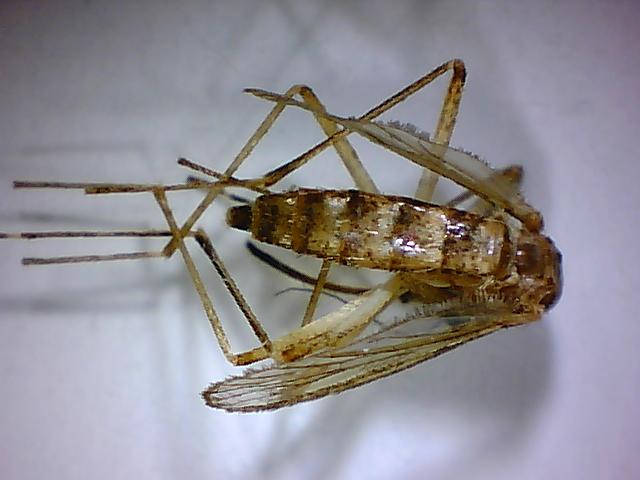


*Aedes vexans*: JHU-000379_05m.jpg, JHU-001016_02m.jpg, JHU-001092_02m.jpg, JHU-001254_01m.jpg


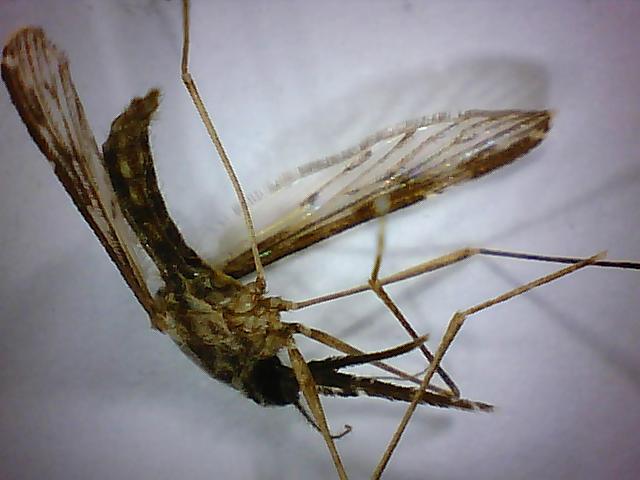

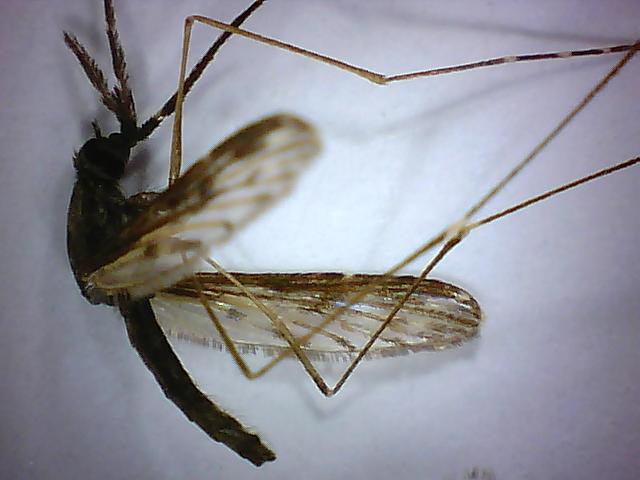


*Anopheles cf-coustani*: JHU-004414_09m.jpg, JHU-004416_03m.jpg


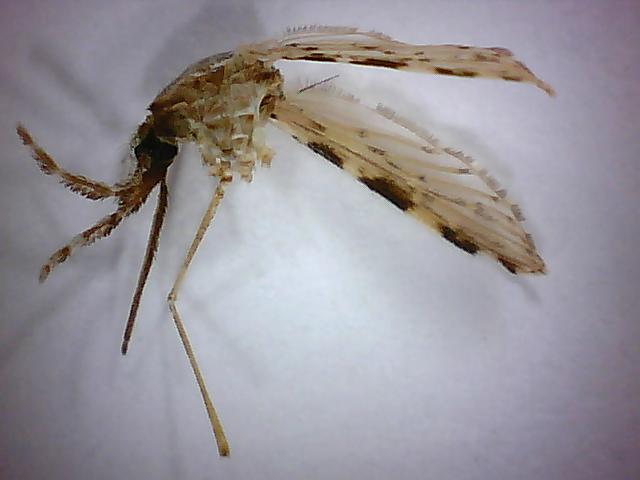

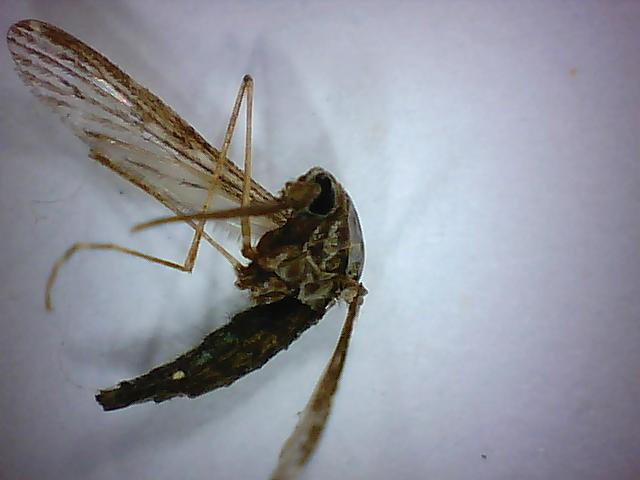

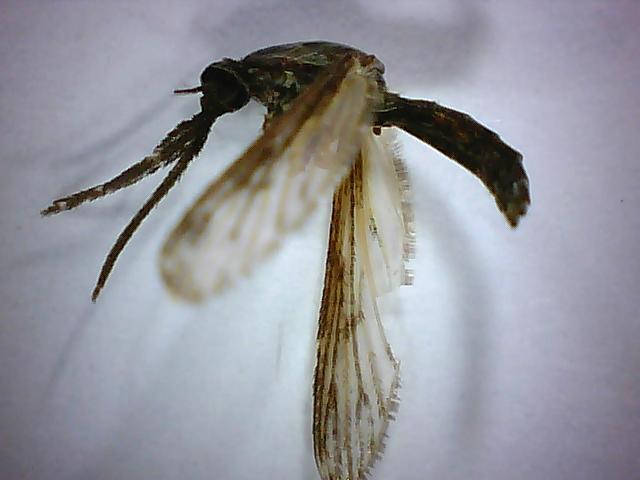

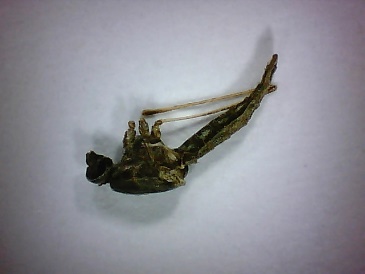


*Anopheles coustani*: JHU-004408_02m.jpg, JHU-004410_04m.jpg, JHU-004420_08m.jpg, JHU-005609_03m.jpg


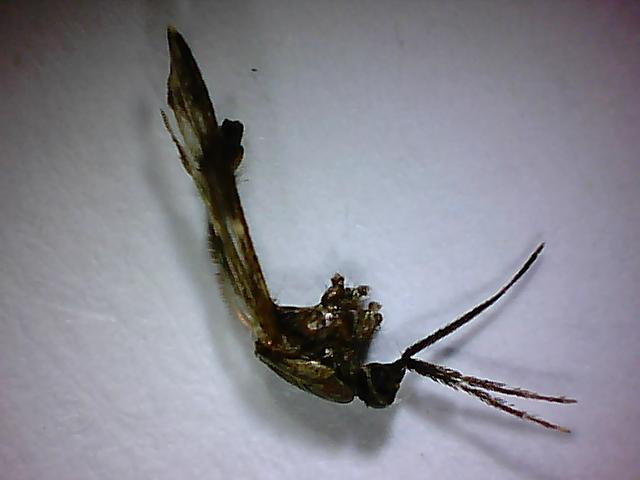

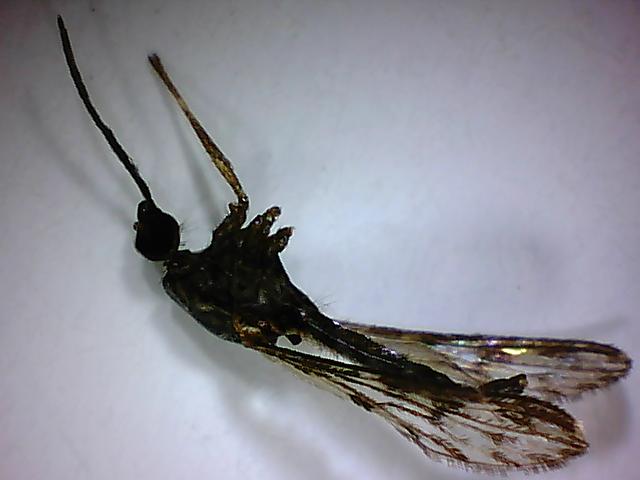

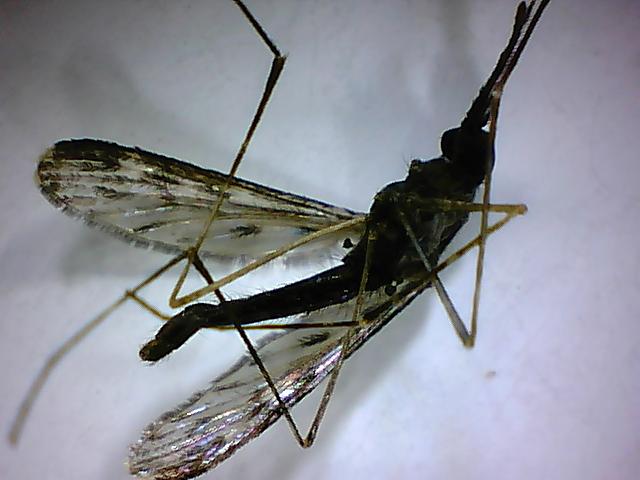

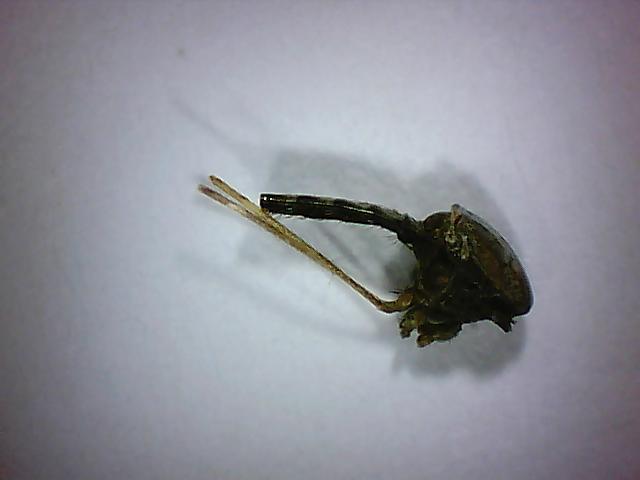


*Anopheles crucians sl*: JHU-000224_04m.jpg, JHU-000508_05m.jpg, JHU-000514_02m.jpg, JHU-002219_05m.jpg


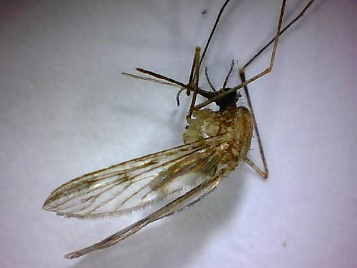

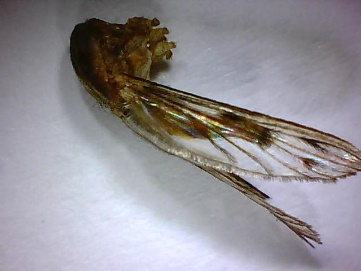

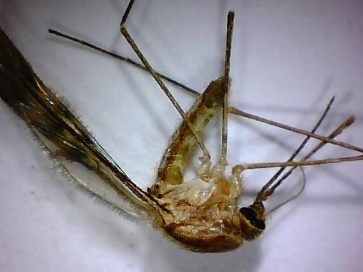

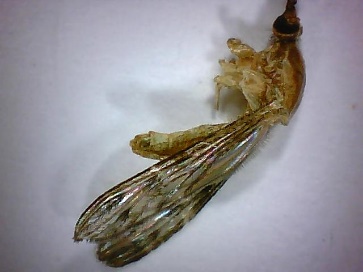


*Anopheles freeborni*: JHU-002287_01m.jpg, JHU-002291_03m.jpg, JHU-002298_05m.jpg, JHU-002357_04m.jpg


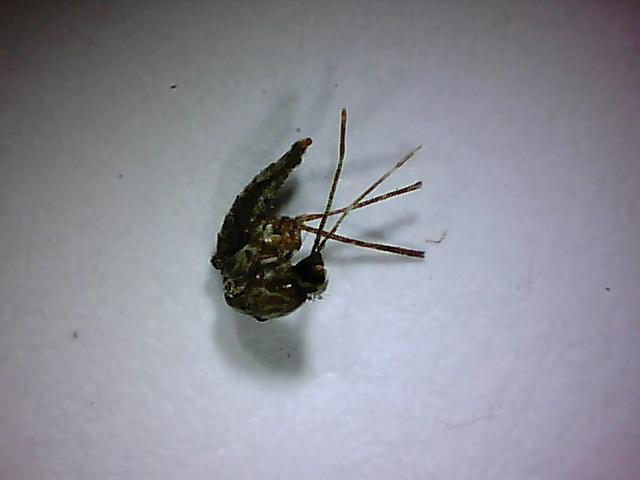

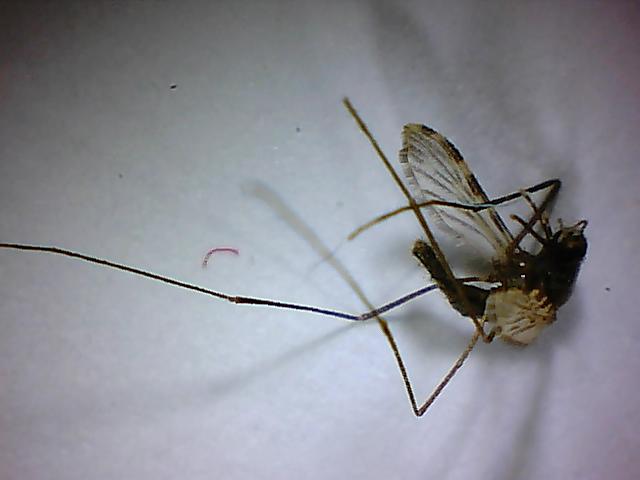

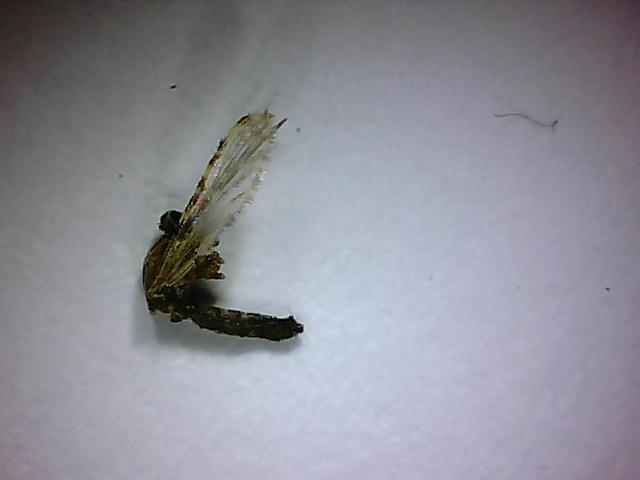

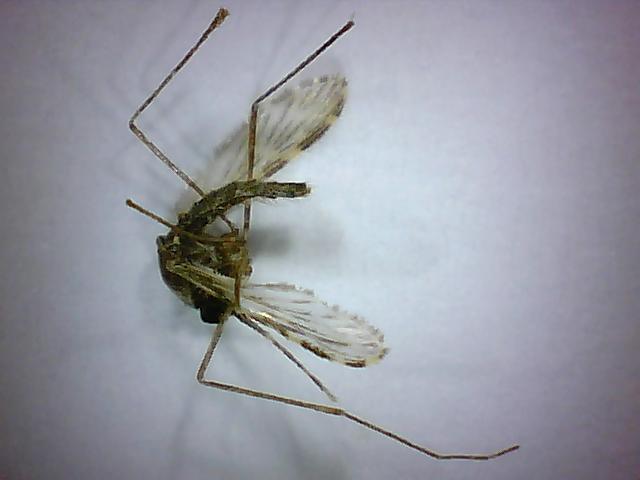


*Anopheles funestus sl*: JHU-004152_01m.jpg, JHU-004191_03m.jpg, JHU-004232_02m.jpg, JHU-004498_02m.jpg


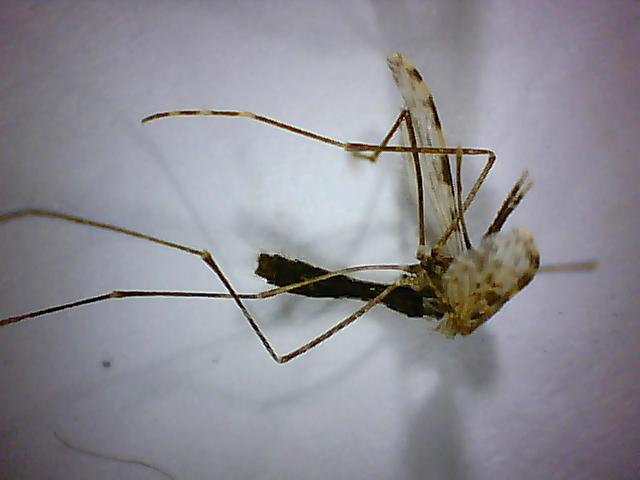

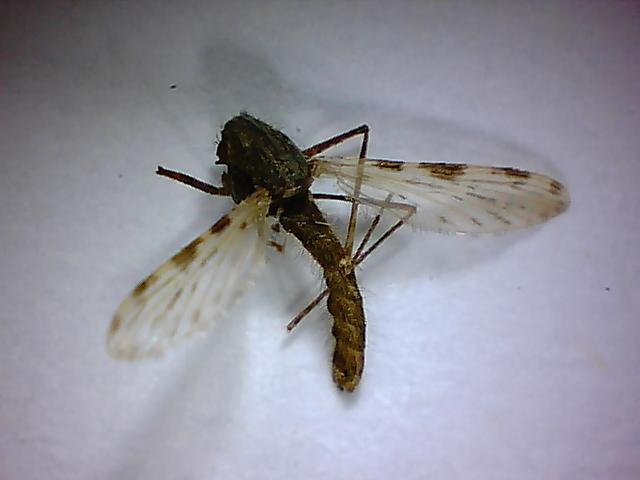

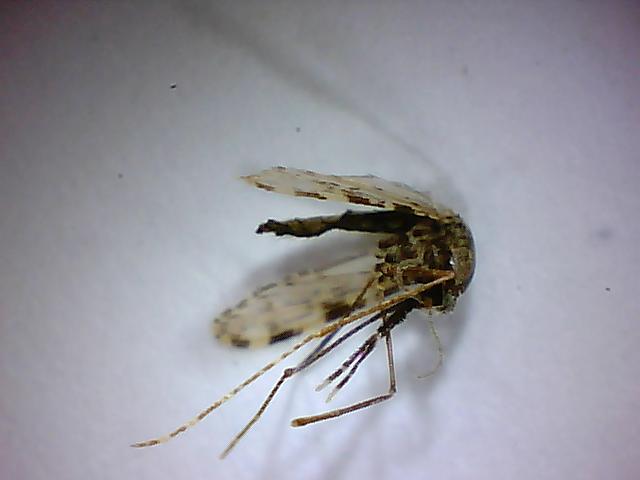

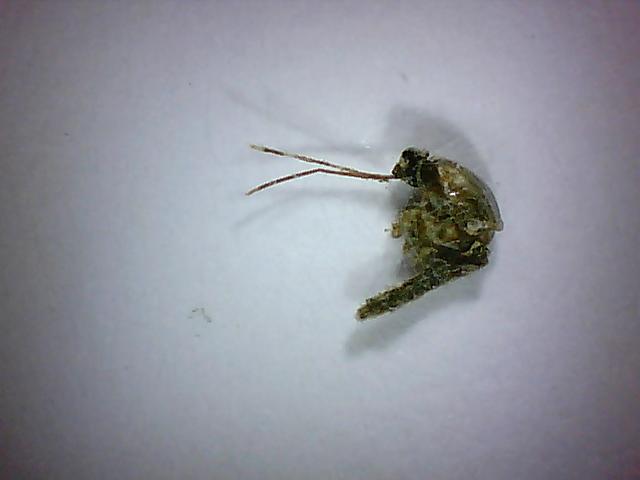


*Anopheles gambiae sl*: JHU-004337_01m.jpg, JHU-004631_01m.jpg, JHU-004606_09m.jpg, JHU-004342_02m.jpg


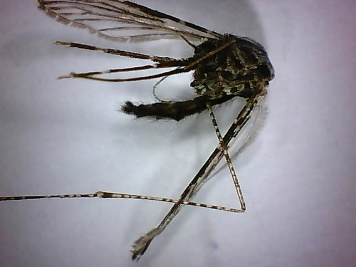


*Anopheles maculipalpis*: JHU-005667_01m.jpg


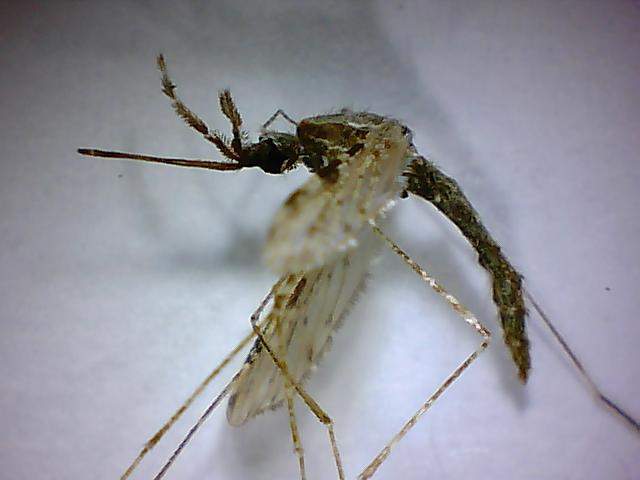


*Anopheles pharoensis*: JHU-004669_06m.jpg


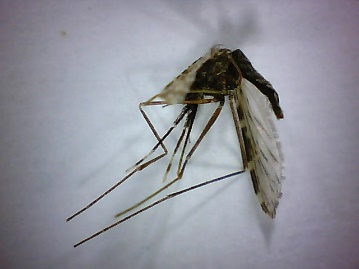


*Anopheles* *pretoriensis*: JHU-005614_04m.jpg


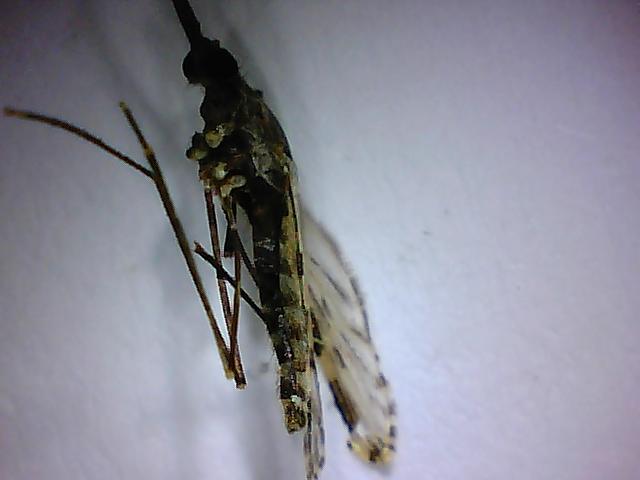


Anopheles pseudopunctipennis: JHU-002281_03m.jpg


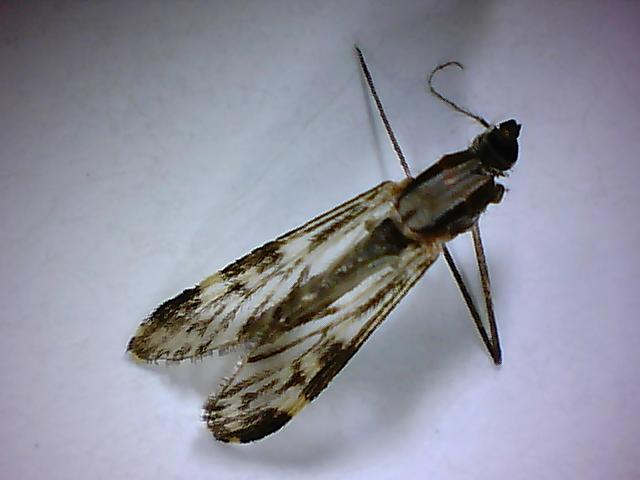

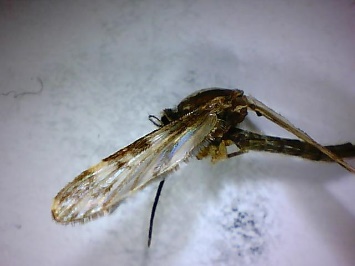

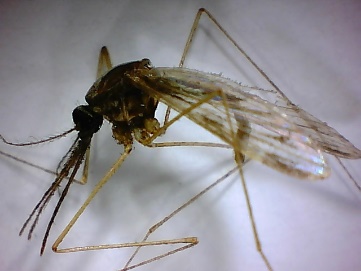

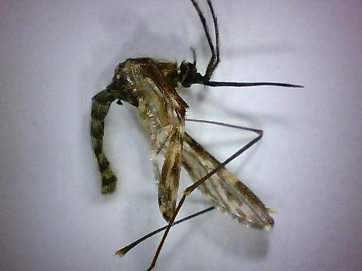


Anopheles punctipennis: JHU-000274_02m.jpg, JHU-001026_03m.jpg, JHU-001218_03m.jpg, JHU-005347_01m.jpg


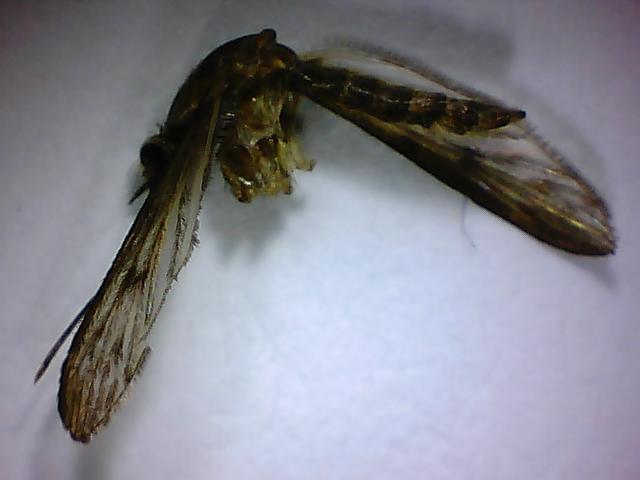

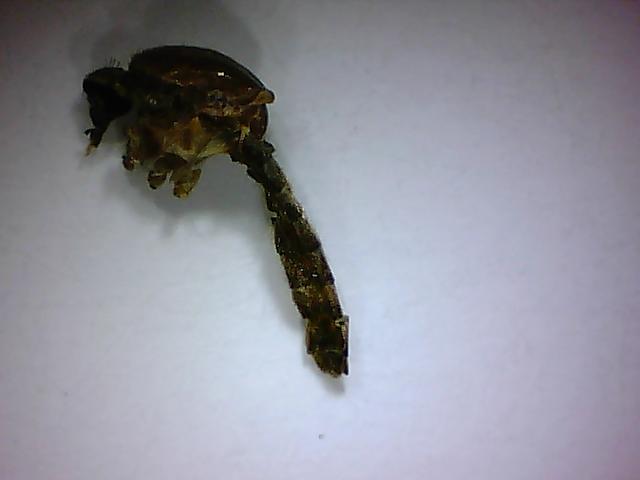

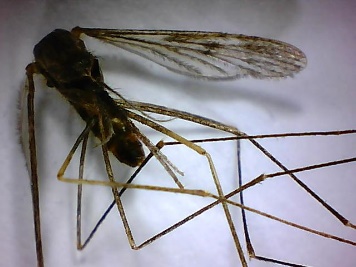

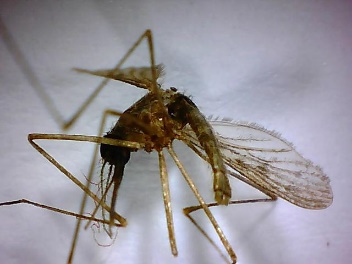


*Anopheles quadrimaculatus*: JHU-000218_02m.jpg, JHU-000375_04m.jpg, JHU-001464_02m.jpg, JHU-001523_01m.jpg


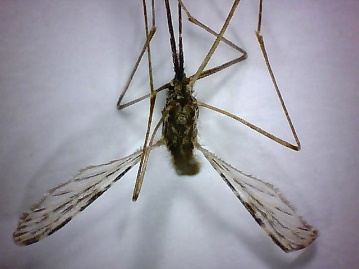


*Anopheles rufipes*: JHU-005593_02m.jpg


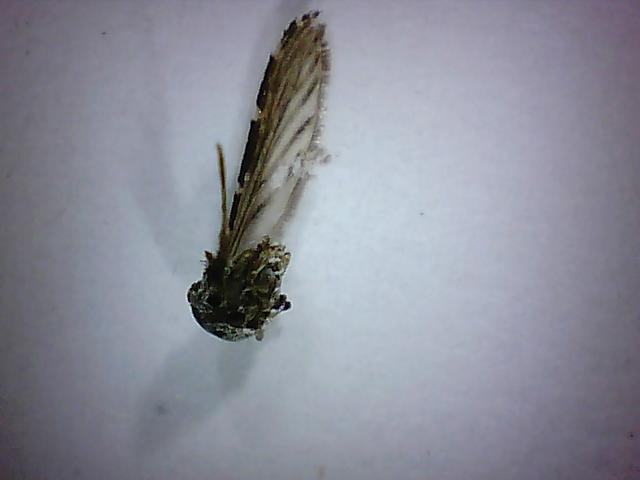


*Anopheles squamosus*: JHU-004418_02m.jpg,


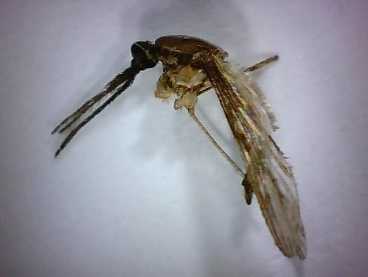


*Anopheles tenebrosus*: JHU-005583_02m.jpg


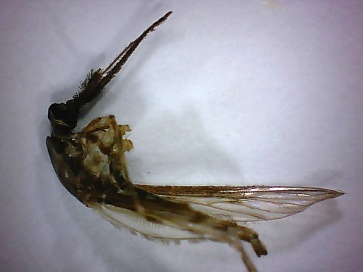

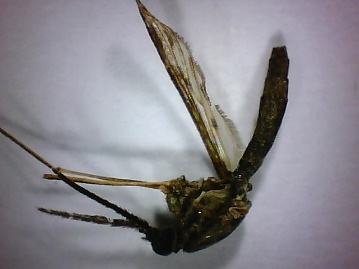


*Anopheles ziemanni*: JHU-005567_04m.jpg, JHU-005575_03m.jpg


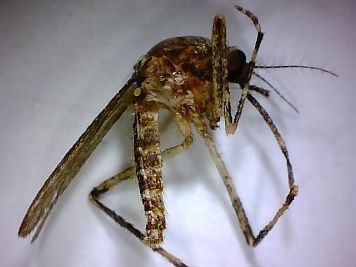

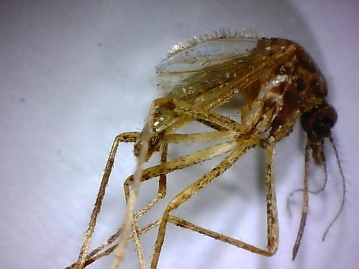


*Coquillettidia perturbans*: JHU-001195_01m.jpg, JHU-001547_06m.jpg, JHU-002203_03m.jpg, JHU-005299_04m.jpg

*Culex antillummagnorum*: JHU-005772_02m.jpg

*Culex bahamensis*: JHU-000360_04m.jpg

*Culex coronator*: JHU-000260_02m.jpg, JHU-000501_02m.jpg

*Culex erraticus*: JHU-000264_05m.jpg, JHU-000309_03m.jpg, JHU-001027_03m.jpg, JHU-001255_03m.jpg

*Culex nigripalpis*: JHU-000513_04m.jpg, JHU-005877_03m.jpg

*Culex pipiens sl*: JHU-000414_03m.jpg, JHU-000426_02m.jpg, JHU-001118_01m.jpg, JHU-005874_05m.jpg

*Culex restuans*: JHU-000184_01m.jpg, JHU-00316_02m.jpg

*Culex salinarius*: JHU-000325_03m.jpg, JHU-000502_01m.jpg, JHU-001234_03m.jpg, JHU-002453_02m.jpg

*Culex tarsalis*: JHU-005349_02m.jpg

*Culex territans*: JHU-001507_06m.jpg

Culiseta incidens: JHU-002116_01m.jpg, JHU-005320_06m.jpg

Culiseta inornate: JHU-002115_01m.jpg, JHU-005302_01m.jpg

Culiseta melanura: JHU-000521_02m.jpg

Deinocerites cancer: JHU-000366_01m.jpg

Deinocerites cuba-1: JHU-000440_04m.jpg, JHU-000474_02m.jpg

*Mansonia titillans*: JHU-000208_01m.jpg, JHU-000528_01m.jpg

*Orthopodomyia signifera*: JHU-001504_01m.jpg

*Psorophora ciliata*: JHU-000650_01m.jpg, JHU-001452_02m.jpg, JHU-001466_08m.jpg

*Psorophora columbiae*: JHU-000196_02m.jpg, JHU-000237_02m.jpg, JHU-001472_04m.jpg, JHU-002147_05m.jpg

*Psorophora cyanescens:* JHU-001940_06m.jpg, JHU-0001944_08m.jpg, JHU-001952_08m.jpg, JHU-002015_01m.jpg

*Psorophora discolor:* JHU-005274_03m.jpg

*Psorophora ferox*: JHU-000297_02m.jpg, JHU-001055_02m.jpg, JHU-001212_03m.jpg, JHU-001391_01m.jpg

*Psorophora howardii*: JHU-001010_03m.jpg

*Psorophora pygmaea*: JHU-000340_03m.jpg, JHU-000483_04m.jpg, JHU-000483_03m.jpg

*Psorophora signipennis*: JHU-005328_03m.jpg

*Uranotaenia sapphirina:* JHU-000430_01m.jpg
